# Supplementary material for: A Simulated Case of Acute Salicylate Toxicity From an Intentional Overdose
Source: MedEdPORTAL. 2018 Feb 12;14:10678. doi: 10.15766/mep_2374-8265.10678 (PMC6342373; doi:10.15766/mep_2374-8265.10678)
Supplement: Supplementary file 1 — A. Simulation Case.docx B. Actor Scripts.docx C. Preparation Assignment.docx D. Introduction to Activity.docx E. Lab and Diagnostic Results.docx F. Treatment Options.docx G. Survey Instrument.docx H. Debriefing Questions and Answers.docx I. Debriefing Session PowerPoint.pptx J. Abbreviated Debriefing Questions and Answers.docx [file mep-14-10678-s001.zip › _Educational_Summary_Report_10678.pdf]

MedEdPORTAL®
The Journal of Teaching and Learning Resources

Original Publication

OPEN ACCESS

A Simulated Case of Acute Salicylate Toxicity From an Intentional Overdose

Richard L. Lammers, MD, Maria L. Sheakley, PhD*, Sean Hendren, MD
*Corresponding author: maria.sheakley@med.wmich.edu

Citation: Lammers RL, Sheakley ML, Hendren S. A simulated case of acute salicylate toxicity from an intentional overdose. *MedEdPORTAL*. 2018;14:10678. https://doi.org/10.15766/mep_2374-8265.10678

Copyright: © 2018 Lammers et al. This is an open-access publication distributed under the terms of the Creative Commons Attribution-Share Alike license.

Abstract

Introduction: Salicylate poisoning is a serious toxicologic problem with a complex pathophysiology that requires prompt diagnosis and action for a favorable outcome. A simulated experience in the evaluation and management of an aspirin-overdose patient allows learners to construct a differential diagnosis from an array of symptoms and signs, analyze a mixed acid-base disturbance, and explore the multistep management of this disorder. **Methods:** This simulation exercise was designed for second-year medical students. At the start of the session, teams received a 10-minute introduction to the activity. Upon entering a room in a simulated Emergency Department, teams had 15 minutes to complete a focused history and physical exam of the patient, interpret arterial blood gas and basic metabolic panel data, and administer treatment based on key findings and a presumptive diagnosis. The scenario was followed by a 90-minute facilitated debriefing session. An alternative 45-minute debriefing guideline is also included. **Results:** Students voluntarily completed a 13-question, 5-point Likert-scale survey about the educational exercise immediately following the session. They evaluated the preparatory materials and briefing, the simulation scenario, the usefulness of the debriefing, and their confidence in their understanding of salicylate poisoning following the session. Students reported a favorable response to the overall experience and the debriefing, as well as an increase in confidence following the session. **Discussion:** This simulation exercise was successful in exposing students to the clinical presentation of salicylate toxicity and giving them the opportunity to apply and synthesize basic science knowledge during the scenario.

Keywords

Simulation, Metabolic Acidosis, High-Fidelity Simulation, Renal Physiology, Salicylate Toxicity, Acid-Base Disturbance, Editor's Choice

Appendices

A. Simulation Case.docx
B. Actor Scripts.docx
C. Preparation Assignment .docx
D. Introduction to Activity .docx
E. Lab and Diagnostic Results .docx
F. Treatment Options.docx
G. Survey Instrument.docx
H. Debriefing Questions and Answers.docx
I. Debriefing Session PowerPoint.pptx
J. Abbreviated Debriefing Questions and Answers .docx

All appendices are peer reviewed as integral parts of the Original Publication.

Educational Objectives

By the end of this session, learners will be able to:

1. Identify the signs and symptoms of acute salicylate toxicity in a simulated patient.
2. Use arterial blood gas and basic metabolic panel data to identify the underlying acid-base disturbances.
3. Explain abnormal physical findings and laboratory values on the basis of pathophysiology.
4. Describe the pharmacologic mechanism of aspirin and its metabolism as it relates to toxicity.
5. Discuss the general treatment goals for a patient with acute salicylate toxicity.

Introduction

The first clinical reports of using willow bark, a natural salicylate-containing substance, to treat fever and pain were made in 1763 by the English clergyman Edward Stone.[1] Sixty-five years later, the active chemicals were isolated from willow bark.[2] Salicylates (i.e., aspirin) are still used today to treat pain, fever, and inflammation.

Isolated salicylate poisoning was the 14th most common cause of death from toxic exposures recorded by the American Association of Poison Control Centers' National Poison Data System in 2014 in the United States.[3] Salicylate overdose remains a significant cause of toxicity and death in part because of easy access to this drug. Unintentional toxicity may occur in patients who are unaware of the presence of

salicylates in over-the-counter medications. Aspirin is frequently used in suicide attempts since it is commonly found in most households. A skilled initial assessment of a patient poisoned with salicylates is essential for accurate diagnosis, determination of severity, and appropriate treatment.

A high-fidelity simulation exercise was designed for second-year medical students by emergency medicine physicians to (1) demonstrate the symptoms and signs of salicylate toxicity, (2) provide practice in diagnosing a mixed acid-base disturbance, and (3) introduce students to various treatment modalities for this poisoning. This exercise allows students an opportunity to work in teams under close faculty supervision in an educationally safe environment. Several salicylate toxicity simulation exercises have been published previously[4,5]; however, this exercise is different in that it requires students to justify their diagnosis and treatment decisions during the debriefing session by applying their knowledge of the underlying pathophysiology and acid-base disturbances.

Methods

Development

Western Michigan University Homer Stryker M.D. School of Medicine (WMed) has an integrated, organ-system-based curriculum. This simulation exercise (Appendix A) occurred during the Renal Course, which is delivered during the second year of the preclinical curriculum. Prior to this event, students had completed the Foundations Courses (Molecular, Cell, Genetics, and Metabolics), as well as Immunology & Infectious Disease, Musculoskeletal, Cardiovascular, and Pulmonary Courses. This simulation session was scheduled near the end of the Renal Course, following most of the learning events on renal function and acid-base disturbances. Sixty students from the WMed class of 2019 completed the simulation exercise as a normal part of the curriculum.

Equiment/Environment

Upon entering the simulated emergency rooms, the teams were introduced to the patient (a mannequin) and a nurse (an embedded participant/actor). Students were allowed to assemble their team, interview and examine the patient, order and interpret tests, and initiate treatment in any manner they chose, without interruption from faculty. If student teams struggled with treatment decisions, the nurse was instructed to provide scripted prompts. These actor scripts are provided in Appendix B.

Personnel

To run the 15-minute simulation session, one clinical faculty member and one simulation technician (nurse actor) were needed per team (three teams were run simultaneously), and one extra simulation technician was present to oversee the whole event. The clinical faculty member played the part of the simulated patient in each scenario (i.e., read the patient script) and operated the mannequins from a control room as treatments were administered. If the faculty member was female, the mannequin was given a female appearance to match the voice. Faculty members also played the role of the Poison Control Center toxicologist, if called. The three simulation technicians acting as nurses (one in each scenario) were instructed to follow scripts, provide information and cues included in those scripts (e.g., a suggestion to “call Poison Control”), provide equipment, and deliver medications and fluids, as requested by the student teams. The additional simulation technician was present in the control room to time the scenarios, make sure the equipment was functioning properly, and assist as needed.

Implementation

This 2-hour simulation session was repeated for four groups of 15 students. No changes were made between each session. One week prior to the event, a prereading assignment was sent to the students to guide their preparation for the simulation exercise (Appendix C). To provide optimal team sizes during the scenarios, each group of 15 students was subdivided into three teams of five students. Identical simulations were conducted simultaneously in three separate rooms.

Sample Schedule of Simulation Events

- 3:00-3:10 pm: introduction to the simulation scenario.
- 3:10-3:25 pm: simulation activity.
- 3:25-3:30 pm: short break to transfer to debriefing classroom.
- 3:30-5:00 pm: debriefing.

At the start of the simulation session, each group of students was given 10 minutes of verbal instructions about functioning in a simulated clinical environment and about the specific simulation activity (Appendix D). They were informed that they would have 15 minutes to evaluate a patient in an Emergency Department, record key clinical findings, order and interpret laboratory studies, and apply their knowledge of acid-base balance to attempt a diagnosis and treatment plan. They were advised to assign roles (team leader, recorder, and support roles) to maximize efficiency.

The teams could order any laboratory test they deemed appropriate. If the test results were available, they were given to the team at the 8-minute time point in the scenario. Not all tests that were requested were available during the scenario. Nevertheless, the teams had access to enough information to make diagnosis and treatment decisions for the patient. The diagnostic labs and tests that were available, if requested, were as follows:

- EKG report from cardiologist.
- Chest X-ray report from radiologist.
- Comprehensive metabolic panel (with normal ranges).
- Complete blood count without differential (with normal ranges).
- Arterial blood gas on room air (with normal ranges).
- Urinalysis (with normal ranges).
- Urine or serum toxicology screens.
- Quantitative serum salicylate level (with normal ranges).
- Serum osmolarity (with normal range).
- Lactic acid (with normal range).

The results of each test listed above can be found in the lab and diagnostic results document (Appendix E). In addition, consultation with a Poison Control Center toxicologist was available, and a variety of simulated procedures and treatments could be administered upon request. If a treatment was ordered, the team was handed an image of it (Appendix F), and the mannequin operator would produce the appropriate response in the simulated patient.

Assessment
We examined the impact of this simulation case on second-year medical student perceptions using a nonrandomized study design for continuous quality improvement. Upon conclusion of the simulation event, students voluntarily completed an anonymous, 13-question evaluation of the entire session (Appendix G). The survey instrument, which was modified from an article by Leighton, Ravert, Mudra, and Macintosh,[6] utilized a 5-point Likert scale and optional comments section. This investigation was approved by the Institutional Review Board of Western Michigan University. Informed consent to participate in this research study was obtained through a verbal description of the protocol to small groups of students and by voluntary completion of a written questionnaire.

Debriefing
At the conclusion of the scenario, all three teams assembled in one classroom for a 90-minute debriefing and discussion session (Appendix H) with a PowerPoint presentation (Appendix I). For programs with reduced curricular time allotment for simulation, a shortened 45-minute debriefing guide is also available (Appendix J). The session was cofacilitated by a clinician, a basic science faculty member (physiologist),

and a clinical pharmacologist. The method of debriefing included the reaction phase, analysis phase, and summary phase. The reaction phase allowed participants to discuss their feelings about the case and decompress from the stress of the event. The analysis phase involved a review of the medical facts of the case, including details of the patient's history and physical exam, lab results, and the diagnostic and treatment regimens. During this phase, the facilitators asked a series of key questions about the patient's history and physical exam, and the cause of each physical finding was discussed to ensure understanding of the underlying physiologic and pharmacologic mechanisms. The facilitator's job was to guide the discussion rather than lead it, encourage participation from everyone, limit interruptions by others when someone was speaking, ensure a confidential and safe environment, and allow time for participants' responses. Finally, in the summary phase, participants reviewed the lessons they had learned about aspirin toxicity in the simulation and during the debriefing and discussion session.

The shortened debriefing guide has less focus on the fundamental acid-base discussion and does not discuss the mechanism of bicarbonate for treating salicylate poisoning. In addition, the instructor can accelerate the first half of the debrief by instructing students to report and discuss only abnormal findings or modify the debrief session to fit the specific needs of a curriculum.

Results

All 60 students voluntarily completed the anonymous survey (Table) immediately following the debriefing. Questions 1-3 referred to the advance preparation assignment and presimulation briefing; questions 4-6 referenced the simulation activity; questions 7-9 referenced the students' confidence following the simulation; and questions 10-13 referred to the debriefing session. In addition, 10 students provided written comments. Comments included the following:

- "Really enjoyed the complexity of this simulation."
- "This was a great exercise. I wish we had more instruction before the simulation."
- "I liked the time taken to relate the simulation to pathophysiology."
- "This was very helpful! Thank you!"
- "This was actually a very enjoyable simulation."
- "This simulation was most valuable in selecting the appropriate treatment."
- "Excellent! Great discussion after simulation."
- "I enjoyed this! It tied together a lot of concepts for me."
- "Having such thorough debriefs with feedback and going over different scenarios is one of the most integral and important parts of our education. It helps me retain important information."
- "Really good simulation! I learned a lot!"

Table. Survey Instrument Used by Students (*N* = 60) to Evaluate the Simulation Session, With Aggregate Responses

Question	Strongly Disagree	Disagree	Neutral	Agree	Strongly Agree
1. Prereading assignments prepared me for the salicylate toxicity simulation activity.	0%	4%	9%	48%	39%
2. Briefing before the simulation was beneficial.	2%	2%	36%	35%	26%
3. Briefing before the simulation increased my confidence.	2%	5%	44%	34%	15%
4. During the simulation, I had the opportunity to practice my clinical decision-making skills.	0%	0%	8%	33%	59%
5. During the simulation, I had the opportunity to experience how time pressure can affect my clinical decision-making skills.	0%	3%	8%	23%	66%
6. During the simulation, I had the opportunity to work as part of a health care team.	0%	2%	3%	18%	77%
7. I am more confident in my ability to report information to my health care team.	0%	4%	6%	51%	39%
8. I am more confident in my understanding of the pathophysiology of salicylate toxicity.	0%	2%	0%	38%	60%
9. I am more confident in my ability to differentiate between different types of acid-base disturbances.	0%	3%	10%	44%	43%
10. Debriefing contributed to my learning.	4%	0%	0%	22%	74%
11. Debriefing was valuable in helping me select the appropriate treatments for salicylate toxicity.	3%	0%	3%	27%	66%
12. Debriefing provided adequate time to review the critical concepts related to salicylate toxicity, including acid-base disturbances.	2%	0%	3%	35%	60%
13. Debriefing provided opportunities to self-reflect on my performance during the simulation.	2%	2%	3%	38%	55%

Discussion

The purpose of this simulation exercise was twofold: first, to expose students to the clinical presentation of salicylate toxicity, and second, to give students the opportunity to apply and synthesize previously learned basic science knowledge during a simulated clinical scenario. Simulation exercises have been shown to improve student self-confidence with real clinical cases and competence in basic clinical skills.[7,8] The goal of this experience was to improve students' confidence when diagnosing and treating salicylate toxicity and other acid-base disturbances during future clerkships and clinical practice.

The development of this simulation was integrated and multidisciplinary. An emergency medicine faculty/clinician and resident attempted to accurately portray the clinical presentation of this disorder by using data from actual patients and by programming the algorithms for the simulator responses to each potential treatment. Following case development, a physiologist and pharmacologist reviewed the scenario to integrate the basic science content. All of these faculty members worked together to develop the debriefing discussion points. Collaboration between clinicians and basic scientists supports curricular integration and well-rounded case development.

The majority of students agreed that the prereading assignment prepared them for the simulation activity (question 1), which was evident in the overall student performance during the scenario and their comments and responses during the debriefing session. In contrast, in response to questions 2 and 3, a relatively large percentage of students had a neutral (or negative) opinion of the briefing that occurred just prior to the start of the activity. Forty percent of students did not agree that the briefing was beneficial (question 2), and 51% did not agree that the briefing increased their confidence (question 3). The briefing before the simulation activity is one part of the activity that can be improved. It is possible that students expected the briefing to provide information that would have improved their performance during the scenario, which was not our intent. The purpose of the briefing was to prepare the students for the simulation by providing clear and concise objectives for the session, introducing the setting and resources available, and relaying expectations. This was typically done verbally, but a short presentation or written keywords may help to better facilitate this session.

Questions 4-6 of the survey focused on the student experience during the simulation activity. Nearly all students responded positively (answering *agree* or *strongly agree*) that simulation provided an opportunity to practice clinical decision-making skills, experience how time pressure can affect decision-making skills, and work as part of a health care team (92%, 89%, and 95%, respectively). In fact, this simulation exercise was designed to provide all of these experiences during the scenario phase, illustrating that simulation is an ideal tool for achieving these objectives.

Questions 7-9 focused on the students' confidence and knowledge of the content. A majority of students responded positively about increased confidence in their ability to report information to a health care team, ability to understand the pathophysiology of salicylate toxicity, and ability to differentiate between different types of acid-base disturbances (90%, 98%, and 87%, respectively). While working through this simulated case, the students were given a safe and realistic setting to apply their theoretical knowledge of renal function and acid-base imbalances. Simulated clinical experiences help bridge the gap between the theory and practice of medicine and assist students in developing a systematic approach to clinical problems.

Questions 10-13 on the survey focused on the debriefing session following the simulation scenario. Nearly all students agreed or strongly agreed that the debriefing contributed to their learning, helped them select appropriate treatments for salicylate toxicity, allowed time to review critical concepts related to salicylate toxicity and acid-base disturbances, and provided an opportunity to self-reflect on their performance during the simulation (96%, 93%, 95%, and 93%, respectively). The debriefing session is a crucial component of simulations, clarifying and consolidating insights and lessons learned, and an opportunity for students to reflect on their actions, thought processes, and emotional states throughout the activity.

10.15766/mep_2374-8265.10678

MedEdPORTAL®
The Journal of Teaching and Learning Resources

Debriefing is where the learning framework is created and contextualization occurs. This debriefing session, as designed and delivered, appears to have accomplished those objectives.

Although this simulation exercise was designed for medical students, it has been easily adapted for resident physician learners who wanted to reinforce the diagnosis and treatment of acid-base disorders. Different preparatory reading material had to be provided to the resident physicians. With some work, this scenario could potentially be adapted for interdisciplinary groups of learners, including pharmacy and nursing students; however, it is not designed for this in its current form. The biggest challenges in this simulation exercise involved (1) scheduling and training faculty and technicians, since multiple personnel were needed to run the event (three clinicians, a clinical pharmacologist, a physiologist, and four simulation technicians), and (2) designing visually engaging and effective debriefing materials to help students walk through the acid-base disturbance in a systematic way.

A limitation of this study is that the evaluation of the exercise was based on self-reports collected from the anonymous 13-question survey. Future studies will include an evaluation of student learning based on summative examination performance on questions relative to the concepts applied in the simulation exercise.

Richard L. Lammers, MD: Professor, Department of Emergency Medicine, Western Michigan University Homer Stryker M.D. School of Medicine; Assistant Dean for Simulation, Western Michigan University Homer Stryker M.D. School of Medicine

Maria L. Sheakley, PhD: Associate Professor, Department of Biomedical Sciences, Western Michigan University Homer Stryker M.D. School of Medicine

Sean Hendren, MD: Clinician, Department of Emergency Medicine, UP Health System—Marquette

Disclosures
None to report.

Funding/Support
None to report.

Ethical Approval
This publication contains data obtained from human subjects and received ethical approval.

References

1. Stone E. An account of the success of the bark of the willow in the cure of agues. *Philos Trans R Soc Lond B Biol Sci.* 1763;53:195-200. https://doi.org/10.1098/rstl.1763.0033
2. Schindler PE Jr. *Aspirin Therapy*. New York, NY: Walker Publishing; 1978.
3. Mowry JB, Spyker DA, Brooks DE, McMillan N, Schauben JL. 2014 annual report of the American Association of Poison Control Centers' National Poison Data System (NPDS): 32nd annual report. *Clin Toxicol (Phila)*. 2015;53(10):962-1147. https://doi.org/10.3109/15563650.2015.1102927
4. Mitzman J, Wittler M. Toxic ingestion: anion gap metabolic acidosis caused by acute salicylate ingestion. *MedEdPORTAL*. 2014;10:9893. https://doi.org/10.15766/mep_2374-8265.9893
5. Ryan M, Beattie L. Acute overdose in the setting of a signed 'do not resuscitate' order. *MedEdPORTAL.* 2014;10:9945. https://doi.org/10.15766/mep_2374-8265.9945
6. Leighton K, Ravert P, Mudra V, Macintosh C. Updating the Simulation Effectiveness Tool: item modifications and reevaluation of psychometric properties. *Nurs Educ Perspect.* 2015;36(5):317-323. https://doi.org/10.5480/15-1671
7. Aboumatar HJ, Thompson D, Wu A, et al. Development and evaluation of a 3-day patient safety curriculum to advance knowledge, self-efficacy and system thinking among medical students. *BMJ Qual Saf.* 2012;21(5):416-422. https://doi.org/10.1136/bmjqs-2011-000463
8. Muniandy RK, Nyein KK, Maujus F. Improving the self-confidence level of medical undergraduates during emergencies using high fidelity simulation. *Med J Malaysia.* 2015;70(5):300-302.

Received: August 1, 2017 | **Accepted:** January 6, 2018 | **Published:** February 12, 2018
