# Supplementary material for: A Simulated Case of Acute Salicylate Toxicity From an Intentional Overdose
Source: MedEdPORTAL. 2018 Feb 12;14:10678. doi: 10.15766/mep_2374-8265.10678 (PMC6342373; doi:10.15766/mep_2374-8265.10678)
Supplement: Supplementary file 1 — A. Simulation Case.docx B. Actor Scripts.docx C. Preparation Assignment.docx D. Introduction to Activity.docx E. Lab and Diagnostic Results.docx F. Treatment Options.docx G. Survey Instrument.docx H. Debriefing Questions and Answers.docx I. Debriefing Session PowerPoint.pptx J. Abbreviated Debriefing Questions and Answers.docx [file mep-14-10678-s001.zip › A._Simulation_Case.docx]

| **Appendix A: MedEdPORTAL Simulation Case**  **SIMULATION CASE TITLE:** A Simulated Case of Acute Salicylate Toxicity from an Intentional Overdose.  AUTHORS: Richard L. Lammers, MD, Maria L. Sheakley, PhD., Sean Hendren, MD. | |
| --- | --- |
| **PATIENT NAME:** Mr. Sal Jones (or Ms. Sally Jones)  **PATIENT AGE:** 23 years old  **CHIEF COMPLAINT:** Vomiting | |
|  | |
| **Brief narrative description of case** | A 23-year-old male is brought to the Emergency Department after vomiting in the dorm room. The patient ingested an uncertain number of aspirin tablets three hours prior to arrival and subsequently presents to the ED with signs of acute salicylate toxicity (nausea, vomiting, tinnitus, tachypnea, hyperthermia, diaphoresis, tachycardia, and agitation). If asked, he (or if the mannequin operator is female, she) will admit that he took the pills in response to suicidal thoughts.  From their preparatory reading, the team should recognize acute salicylate toxicity based on history and physical findings. The team will be prompted by the nurse to call a Poison Control Center. The Center will advise the team about tests and treatment. The nurse will carry out all orders, including the administration of a sodium bicarbonate solution as described by the toxicologist and prepared by a pharmacist. Cardiac monitoring should be started and IV access obtained. Laboratory studies confirm salicylate toxicity with a mixed respiratory alkalosis and an anion gap metabolic acidosis. |
| **Primary Learning Objectives** | By the end of this session, learners will be able to:   1. Identify the signs and symptoms of acute salicylate toxicity in a simulated patient. 2. Interpret arterial blood gas and basic metabolic panel to identify the underlying acid-base disturbances. 3. Explain abnormal physical findings and laboratory values on the basis of pathophysiology. 4. Describe the pharmacologic mechanism of aspirin and its metabolism as it relates to toxicity. 5. Discuss the general treatment goals for a patient with acute salicylate toxicity. |
| **Critical Actions** | 1. Assign roles to each team member before entering the patient room, ensuring that someone is assigned the role of scribe and another serves as team leader. 2. Complete a focused history and physical exam. 3. Identify clinical findings consistent with salicylate toxicity, including vomiting, diaphoresis, hyperthermia, tachycardia, tachypnea, and tinnitus. 4. Determine that the patient has salicylate toxicity. 5. Call poison control for treatment instructions. 6. Order IV normal saline and IV bicarbonate solution as treatment. |
| **Learner Preparation** | The learners should have an understanding of renal physiology and acid-base disturbances before this simulation activity. The two preparation assignments below discuss salicylate toxicity and acid-base disorders.  **Reference #1:** Yip L. Aspirin and Salicylates. In: Tintinalli JE, Stapczynski J, Ma O, Cline DM, Cydulka RK, Meckler GD, T. eds. Tintinalli's Emergency Medicine: A Comprehensive Study Guide. New York, NY: McGraw-Hill; 2011.  *Read these sections: Pathophysiology, Clinical features, Diagnosis, Treatment.*  **Reference #2:** Nicolaou DD, Kelen GD. Acid-Base Disorders. In: Tintinalli JE, Stapczynski J, Ma O, Cline DM, Cydulka RK, Meckler GD, T. eds. Tintinalli's Emergency Medicine: A Comprehensive Study Guide. New York, NY: McGraw-Hill; 2011.  *Read these sections: Acid-Base Disorders: Introduction, Measurement of Plasma Acidity, Physiology of Acid Production and Excretion, Fundamental Acid-Base Disorders, Metabolic Acidosis, Clinical Approach to Acid-Base.*  **Reference #3:** See required terminology and a summary of the pathophysiology of salicylate toxicity in appendix B. |

| Initial Presentation | | | |
| --- | --- | --- | --- |
| **Initial vital signs** | BP 110/76  Pulse 118  Respiratory rate 28  Temp 38.0^o^ C  Sp0_2_ 100%  Weight 70 Kg | | |
| **Overall Appearance** | Upon entry to the room, the learners will see a fully dressed patient who is lying in bed at a 45-degree angle. He is awake and oriented but is mildly agitated. He is diaphoretic. The patient vomits periodically but is able to protect his airway, and there is a towel on the patient with emesis on it, and a basin with emesis. He has slight difficulty hearing questions due to tinnitus. His examination is notable for tachypnea, tachycardia, and hyperthermia. No monitors are on the patient. | | |
| **Actors and roles in the room at case start** | A nurse at the bedside introduces the patient and awaits instructions. During the scenario, the nurse provides further scripted information, pre-planned cues, diagnostic test results, and requested equipment. The nurse will describe physical findings that cannot be portrayed by the mannequin while staying in role. The nurse performs only those interventions requested by the learners. The nurse troubleshoots equipment and attempts to mitigate simulation artifacts that interfere with the case. The nurse receives instructions through an earpiece from an instructor in the Control Room, as needed. A simulation technician or other health care provider with basic medical knowledge (eg. EMT level) and who is familiar with the capabilities of the mannequin can play this role.  Nurse Script:  *Scenario time = 0 mins:*  “I’m the nurse taking care of the patient.  Are you the team who’s assigned to him?  His name is Mr. Sal Jones (or Ms. Sally Jones).  He was brought here by his dorm roommate because he’s been vomiting for the past few hours. The roommate thinks he took an overdose of pills.”  *Scenario time = 4 mins; if team does not order labs or treatment:*  “What would you like me to do, doctors?”  “Would you like me to order any tests to see if he has a poisoning?”  “You could call the Poison Control Center for some advice.”  *Scenario time = 8 mins; if lab tests are ordered:*  “Here are the results of the tests you ordered.”  (Provide all results and reports.)  *Scenario time = 10 mins; if sodium bicarbonate infusion is ordered:*  “The pharmacist will make the solution if you tell him how much you want."  *Scenario time = 15 mins, or when the team transfers care to the admitting physician:* “Doctors, the admitting physician is here to take over the care of the patient.”  A faculty instructor is present in the Control Room. This person serves as the voice of the patient and Poison control operator, operates the computer by triggering manual changes as scripted, guides the nurse/actor by direct-talk two-way radio, and terminates the scenario at fifteen minutes. The faculty instructor observes the performance of the team, provides feedback, and co-facilitates the debriefing session. | | |
| **HPI** | At the start of the scenario, the nurse tells the learners the following:   - Patient name: Mr. Sal Jones (or Ms. Sally Jones) - Weight: 70 Kg - Demographics: 23 y/o male (or female) - ED arrival information: driven by roommate - Chief complaint: vomiting - Significant history/details: possible aspirin overdose   The following Information will be provided by the patient, if requested:   - Allergies: NKDA - Home medications: none - Medical history: none - Surgical history: none - Social history: no smoking - Family comments: none - Burning pain in his upper abdomen (epigastrium) - Ringing in his ears - Feeling breathless - Feelings of “stress” and depression - If asked anything about suicidal thoughts or attempts to harm himself, he will admit that he took “handfuls” of pills in response to suicidal thoughts. He also states one time that he still wants to die. - If questioned about the number of pills he ingested, the patient is unsure and cannot provide an estimate. - If asked about the type of pills, the patient will pull the bottle of non-enteric coated aspirin from his pocket and give it to the team. The dosage of the pills was 325 milligrams. - If asked, the patient will admit that the bottle was full before the ingestion. Forty pills are missing from the bottle. - The patient explicitly denies taking any medications, co-ingestions, drugs or alcohol. | | |
| **Past Medical/Surgical History** | **Medications** | **Allergies** | **Family History** |
| Depression | None | No known allergies | Not known |
| **Physical Examination** | | | |
| **General** | Diaphoretic, patient vomits periodically but protects airway; breathing rapidly | | |
| **HEENT** | Pupils 4 mm bilaterally and reactive | | |
| **Neck** | No jugular venous distention; trachea midline | | |
| **Lungs** | Clear to auscultation bilaterally, tachypneic | | |
| **Cardiovascular** | Tachycardic, regular rhythm, no murmurs, rubs, or gallops | | |
| **Abdomen** | Soft, non-tender | | |
| **Neurological** | CN 2-12 intact, 5/5 strength in all extremities, normal sensation to light touch in all extremities | | |
| **Skin** | No rash or erythema; diaphoretic | | |
| **GU** | Normal | | |
| **Psychiatric** | Alert and oriented, patient restless and intermittently agitated; slight delay in responses to questioning | | |

| Instructor Notes - Changes and CASE Branch Points | | |
| --- | --- | --- |
| Intervention / Time point | Change in Case | Additional Information |
| *Scenario time = 0 minutes*  Initial patient presentation and briefing from nurse | None | None |
| IV Saline may be administered at any time point in the scenario | HR decreases 5 per min x 6 mins  BP increases 3/2 per min x 6 mins | None |
| *Scenario time = 4 minutes*  Nurse prompts group to order standard lab tests and call poison control, if not already done. | None | Groups should order lab tests and call poison control |
| *Scenario time = 8 minutes*  Lab tests arrive | None | Groups should interpret laboratory studies |
| *Scenario time = 10 minutes*  Treatment with IV sodium bicarbonate 1-2 mEq/kg | RR decreases 1 per min x 6 mins  Operator triggers vomiting once | None |
| *Scenario time = 15 minutes*  Admission to inpatient medicine team | End scenario | Groups move to debrief room |

**Ideal Scenario Flow**

- Cardiac monitor
- IV access
- IV fluids
- IV bicarbonate

The learners enter the room to find a patient who has been vomiting for a few hours. On questioning, they find he is suicidal and took a handful of pills. He tells them the pill bottle is in his pocket and it was full before he took any. The learners should count the remaining pills and calculate the dose he ingested, based on the label information. They place the patient on a cardiac monitor and check his vital signs, and find him to be tachycardic, tachypneic, and hyperthermic. The group should order labs (comprehensive metabolic panel, arterial blood gas, urinalysis, and serum salicylate level), and use the results to determine that the patient has salicylate toxicity with a mixed respiratory alkalosis and an anion gap metabolic acidosis. The group should also call the Poison Control Center. The Poison Control Center toxicologist will recommend intravenous fluids and a dose of 1-2 mEq/kg sodium bicarbonate IV bolus followed by a continuous infusion of 150 mEq in 5% Dextrose in Water (D_5_W) infused at 200 mL/hr. In addition, whole bowel irrigation with a polyglycol solution will be suggested to prevent further absorption because concretions of salicylate can form. The learners should start IV saline, then ask a pharmacist to provide a bicarbonate solution with a recommended infusion rate. The team should recognize that the bicarbonate is used to enhance salicylate excretion by the kidneys rather than to correct the metabolic acidosis. The scenario will end in 15 minutes.

**Anticipated Management Mistakes**

1. *Failure to take notes about treatment when calling the Poison Control Center: All of the groups called poison control during the activity, but many did not write down the instructions, and they did not remember them accurately. This lead to treatment errors, such as administering oral charcoal, even though the toxicologist states that this is ineffective if administered more than 1-2 hours after ingestion of non-enteric coated salicylate (and the patient is at 3-4 hours post ingestion). There were calculation errors when students attempted to prepare the sodium bicarbonate solution themselves. Students should request the help of a pharmacist.*
