# Supplementary material for: A Simulated Case of Acute Salicylate Toxicity From an Intentional Overdose
Source: MedEdPORTAL. 2018 Feb 12;14:10678. doi: 10.15766/mep_2374-8265.10678 (PMC6342373; doi:10.15766/mep_2374-8265.10678)
Supplement: Supplementary file 1 — A. Simulation Case.docx B. Actor Scripts.docx C. Preparation Assignment.docx D. Introduction to Activity.docx E. Lab and Diagnostic Results.docx F. Treatment Options.docx G. Survey Instrument.docx H. Debriefing Questions and Answers.docx I. Debriefing Session PowerPoint.pptx J. Abbreviated Debriefing Questions and Answers.docx [file mep-14-10678-s001.zip › B._Actor_Scripts.docx]

**Appendix B: Actor Scripts**

**Nurses’ Instructions**

***Setting:*** *Emergency Department exam room*

***Clothing:*** *Scrubs, name tag*

**Performance Rules:**

1. Do not provide any information or cues except as scripted.
2. Do not suggest any history or physical examination. Step back and let the team evaluate the patient.
3. If physical findings are unclear because of simulation artifact, you may describe what you see, if asked.
4. You do not have any more information about the patient’s circumstances prior to his arrival.
5. Do not speak for the patient.
6. Get any equipment or medications requested by the team.
7. Attach oxygen tubing, turn it on, apply a face mask, turn on suction, remove the patient’s pants, start an IV line, deliver drugs in doses specified by the team, provide available lab results, and help with anything they need, but ONLY if requested. The nurse will deliver the bicarbonate solution if the team requests the pharmacist to provide it.
8. Provide only those lab and radiograph reports that were ordered—not everything available to you.
9. Read the script away from the bedside to start the scenario.
10. Medical consultants are available (by phone).
11. If you are unclear about how to respond to a question or a request, signal the Control Room by putting your elbow in your hand, and tap the side of your head with your finger. The instructor will give you directions through the earpiece.

**Patient Script**

History of Present Illness:

*What is wrong?* I can’t stop vomiting.

*Do you have diarrhea?* No.

*Do you have pain?* I have a burning pain in my stomach (point to epigastric area).

*How long have you been sick?* Since I took those pills about 3 or 4 hours ago.

*What pills did you take?* Only the ones in this bottle. (Provide bottle.)

*How many did you take?* A few handfuls. The bottle was full when I started to take them.

*Did you take anything else, such as medications, street drugs, or alcohol?* No.

*Why did you take them?* I want to die. I’m having problems at school and with some relationships. I’ve been stressed. I feel depressed. I don’t want to talk about it.

*Do you still want to harm yourself?* Yes. I don’t want to be here.

*Is anything else wrong with you?* Nausea; ringing in both ears; hearing is muffled; sensation of shortness of breath; having trouble concentrating and feel a little confused.

Review of Systems:

All other systems are negative

Medications & Allergies:

None

Medical & Surgical History:

Negative

Physical Examination:

Present a normal exam, except intermittently agitated & restless, slight delay in response to questions (abdomen is diffusely tender.)

**Nurse Script**

*Scenario time = 0 mins:*

“I’m the nurse taking care of the patient in bed #___.

Are you the team who’s assigned to him/her?

His name is Mr. Sal Jones (or, her name is Ms. Sally Jones.)

He/she was brought here by his/her dorm roommate because he/she’s been vomiting for the past few hours. The roommate thinks he/she took an overdose of pills.”

*Scenario time = 4 mins; if team does not order labs or treatment:*

“What would you like me to do, doctors?”

“Would you like me to order any tests to see if there was a poisoning?”

“You could call the Poison Control Center for some advice.”

*Scenario time = 8 mins; if lab tests are ordered:*

“Here are the results of the tests you ordered.”

(Provide all results and reports.)

*Scenario time = 10 mins; if sodium bicarbonate infusion is ordered:*

“The pharmacist will make the solution if you tell him what you want.”

*Scenario time = 15 mins, or when the team transfers care to the admitting physician:*

“Doctors, the admitting physician is here to take over the care of the patient.”

**Poison Control Center Staff Script**

“This is Dr. Smith. I’m a toxicologist at the Poison Control Center. What is the nature of the poisoning?”

“Here’s what you need to know about salicylate poisoning:

A dose of less than 150 mg/kg is minimally toxic. Expect moderate toxicity in the range of 150-300 mg/kg. 300 mg/kg is severely toxic.

When you get a serum salicylate level back, here’s how to interpret it.

The therapeutic range of salicylate is 10-30 mg/dL; early signs of toxicity are seen at 40-50 mg/dL; and >100 mg/dL (or serious signs) is an indication for hemodialysis. If the patient has serious signs of toxicity, that’s also a reason for hemodialysis. *(Patient’s serum salicylate level is 80 mg/dL.)*

There are four treatment principles to keep in mind.

1. Give plenty of IV fluids.

2. Decontaminate the gut with oral charcoal, unless it’s been more than 1 or 2 hours since the ingestion. Then, you could empty the GI tract with whole bowel irrigation.

3. Alkalinize the urine with an IV infusion of sodium bicarbonate. Infuse it at 200 mL/hr. A pharmacist can create the solution for you.

4. Decide if you need to send the patient for dialysis.”
