# Supplementary material for: A Simulated Case of Acute Salicylate Toxicity From an Intentional Overdose
Source: MedEdPORTAL. 2018 Feb 12;14:10678. doi: 10.15766/mep_2374-8265.10678 (PMC6342373; doi:10.15766/mep_2374-8265.10678)
Supplement: Supplementary file 1 — A. Simulation Case.docx B. Actor Scripts.docx C. Preparation Assignment.docx D. Introduction to Activity.docx E. Lab and Diagnostic Results.docx F. Treatment Options.docx G. Survey Instrument.docx H. Debriefing Questions and Answers.docx I. Debriefing Session PowerPoint.pptx J. Abbreviated Debriefing Questions and Answers.docx [file mep-14-10678-s001.zip › C._Preparation_Assignment.docx]

**Appendix C: Preparation Assignment**

**1. Learn these terms:**

- AKA - alcoholic ketoacidosis
- Anion Gap (or AG) - calculation of difference between cations and anions
- Diaphoresis - sweating
- DKA - diabetic ketoacidosis
- Emesis - vomiting
- Enteric Coating - protective coating to delay medication breakdown in stomach
- Hyperpnea - fast breathing
- Hyperpyrexia - elevated temperature
- Hyperthermia - elevated temperature
- Tachycardia - elevated heart rate
- Tachypnea - elevated respiratory rate
- Tinnitus - sound in one ear or both ears, such as buzzing, ringing, or whistling, occurring without an external stimulus and usually caused by a specific condition, such as an ear infection, the use of certain drugs, a blocked auditory tube or canal, or a head injury
- Toxicity - poisoning

**2. Pathophysiology of Salicylate Toxicity: A summary**

Acetylsalicylic acid (Aspirin) is a salicylate drug frequently used as an antipyretic, anti-platelet, anti-inflammatory, and mild analgesic agent. Once ingested into the gastrointestinal tract, it is absorbed by the gut (predominantly the jejunum and to a lesser extent, the stomach and duodenum*)* into the bloodstream. Within the bloodstream, aspirin is rapidly hydrolyzed to salicylic acid (salicylate). Peak salicylate levels within the blood in therapeutic dosing generally occurs in 15-60 minutes; however, the peak concentration can be delayed up to 35 hours in an overdose setting. As the normal hepatic detoxification of salicylate becomes saturated following an overdose, elimination shifts towards the slower renal excretion, and drug half-life increases from 2 to 4 hours to as long as 30 hours (typically around 6 hours).

Salicylates uncouple oxidative phosphorylation in the mitochondria. This inhibits various enzymes of the Krebs cycle, which results in anaerobic metabolism and the production of organic acids, keto-acids, and lactic acid. The accumulation of these components produces the characteristic anion gap metabolic acidosis of salicylate toxicity. Tachypnea (rapid respirations) and hyperpnea (deep respirations) occur in an attempt to compensate for the metabolic acidosis. As the blood becomes more acidic, more salicylate crosses the blood brain barrier, causing nausea and vomiting by stimulation of the chemoreceptor trigger zone in the medulla and mental status changes such as delirium, agitation, lethargy, and ultimately seizures and coma. Nausea and vomiting may also occur as a result of direct gastric mucosal irritation and cyclo-oxygenase inhibition. Uncoupling of oxidative phosphorylation in the mitochondria also affects the electron transport chain and results in hyperpyrexia (fever). The proton gradient of the electron transport chain cannot be effectively used to create ATP and instead creates heat. Hyperpyrexia is often indicative of significant toxicity.

Salicylates directly stimulate the respiratory center of the medulla in the brain, causing hyperventilation and increasing the respiratory excretion of CO_2_, which produces a “primary” respiratory alkalosis. The metabolic acidosis in salicylate toxicity results from an accumulation of a variety of acids—another primary process.

The body compensates for pH abnormalities by neutralizing acid and by increasing the respiratory rate (thereby excreting carbon dioxide.) Bicarbonate is “consumed” in an attempt to mitigate the acidic shift in the serum. Respirations are increased to eliminate the resulting CO_2_, which results in a “compensatory” respiratory alkalosis.


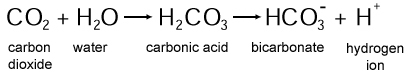


Consequently, there may be multiple primary acid-base disturbances at the same time. The metabolic changes are slow, while the compensatory respiratory changes are rapid. Compensatory mechanisms seldom completely reverse the disorder, and never overcompensate. Remember that “acidemia” and “alkalemia” are measures of the overall pH of the blood and provide no specific information about the specific disorder (described as an acidosis or alkalosis) or the compensatory mechanisms.

**3. Additional Reading – a clinical perspective on acid/base balance:**

**Reference #1:**

Yip L. Aspirin and Salicylates. In: Tintinalli JE, Stapczynski J, Ma O, Cline DM, Cydulka RK, Meckler GD, T. eds. Tintinalli's Emergency Medicine: A Comprehensive Study Guide. New York, NY: McGraw-Hill; 2011.

*Read these sections: Pathophysiology, Clinical features, Diagnosis, Treatment.*

**Reference #2:**

Nicolaou DD, Kelen GD. Acid-Base Disorders. In: Tintinalli JE, Stapczynski J, Ma O, Cline DM, Cydulka RK, Meckler GD, T. eds. Tintinalli's Emergency Medicine: A Comprehensive Study Guide. New York, NY: McGraw-Hill; 2011.

*Read these sections: Acid-Base Disorders: Introduction, Measurement of Plasma Acidity, Physiology of Acid Production and Excretion, Fundamental Acid-Base Disorders, Metabolic Acidosis, Clinical Approach to Acid-Base.*
