# Supplementary material for: A Simulated Case of Acute Salicylate Toxicity From an Intentional Overdose
Source: MedEdPORTAL. 2018 Feb 12;14:10678. doi: 10.15766/mep_2374-8265.10678 (PMC6342373; doi:10.15766/mep_2374-8265.10678)
Supplement: Supplementary file 1 — A. Simulation Case.docx B. Actor Scripts.docx C. Preparation Assignment.docx D. Introduction to Activity.docx E. Lab and Diagnostic Results.docx F. Treatment Options.docx G. Survey Instrument.docx H. Debriefing Questions and Answers.docx I. Debriefing Session PowerPoint.pptx J. Abbreviated Debriefing Questions and Answers.docx [file mep-14-10678-s001.zip › D._Introduction_to_Activity.docx]

**Appendix D: Introduction to Activity**

1. Your objectives during the simulation:
   1. Identify and record patient symptoms and signs for discussion later.
   2. Interpret laboratory studies.
   3. Use staff resources to figure out how to manage the patient.
   4. Use what you have learned about acid/base balance to make sense of this case. (You will benefit from this experience even if you don’t manage the case correctly.)
2. Logistics:
   1. 10-minute orientation
   2. 15-minute simulation
   3. 90-minute (or modified 45 minute) group discussion
3. Rules of confidentiality & professionalism apply. Don’t discuss case or share materials with classmates who have not experienced the case.
4. Setting: an Emergency Department resuscitation room
5. Patient: SimMan 3G (full set of features that you are familiar with.) The patient will interact with you.
6. One actor (nurse) in each room will assist you; interact with this person as you would a real nurse. You can interact with a Poison Control Center pharmacist by phone, if you like.
7. You will work in teams of 5 students per patient.
8. You decide on team assignments, but at least one person should take notes (i.e. the medical record) for the discussion.
9. Stay in your roles. Save your questions for the discussion session. We won’t interrupt the simulation unless your safety is at risk.
10. Keep in mind the time limitation. We will end the scenario at 15 minutes, whether you finish early or don’t complete everything you want to do. You may run out of time if you pursue non-essential tasks or fail to allocate your resources wisely.
11. Enjoy the experience.
