# Supplementary material for: A Simulated Case of Acute Salicylate Toxicity From an Intentional Overdose
Source: MedEdPORTAL. 2018 Feb 12;14:10678. doi: 10.15766/mep_2374-8265.10678 (PMC6342373; doi:10.15766/mep_2374-8265.10678)
Supplement: Supplementary file 1 — A. Simulation Case.docx B. Actor Scripts.docx C. Preparation Assignment.docx D. Introduction to Activity.docx E. Lab and Diagnostic Results.docx F. Treatment Options.docx G. Survey Instrument.docx H. Debriefing Questions and Answers.docx I. Debriefing Session PowerPoint.pptx J. Abbreviated Debriefing Questions and Answers.docx [file mep-14-10678-s001.zip › E._Lab_and_Diagnostic_Results.docx]

**Appendix E: Lab and Diagnostic Results**

1. EKG - Cardiologist report:

Sinus tachycardia; ventricular rate = 120; otherwise normal

2. Chest X-ray – Radiologist report:

Heart & lungs are normal. Mediastinal and hilar structures are normal. Bones and soft tissues are normal. Conclusion: Normal chest film.

**3. Comprehensive Metabolic Panel (CMP)**

Na: (sodium) 141 mEq/L (135-144 normal range)

K (potassium): 3.8 mEq/L (3.7-5.2 normal range)

Cl (chloride): 101 mmol/L (101-111 normal range)

**CO2 (bicarbonate): 12 mmol/L (22-28 normal range; use 24 as normal)**

**BUN (blood urea nitrogen): 25 mg/dL (7-20 normal range)**

Cr (creatinine): 1.3 mg/dL (0.8-1.4 normal range)

Glucose: 85 mg/dL (64-128 normal range)

AST (aspartate aminotransferase): 40 U/L (8-48 normal range)

ALT (alanine aminotransferase): 42 U/L (7-55 normal range)

Alkaline phosphatase: 99 U/L (45-115 normal range)

Total bilirubin: 0.8 mg/dL (0.1-1 normal range)

Albumin: 4 g/dL (3.5-5 normal range)

**4. Complete Blood Count (CBC) without differential**

White blood cells: 9.2 billion cells/L (3.5-10.5 normal range)

Hemoglobin: 14.0 g/dL (13.5-17.5 normal range)

Hematocrit: 42% (38.8-50%)

Platelets: 350 billion/L (150-450 normal range)

**5. Arterial Blood Gas (ABG) on room air**

**pH: 7.31 (7.35-7.45 normal range)**

**PCO2: 22 mm Hg (33-45 normal range)**

PO2: 95 mm Hg (80-100 normal range)

**6. Urinalysis**

Specific gravity: 1.010 (1.005-1.025 normal range)

Color: yellow

pH: 5.5 (4.5-8 normal range)

**Ketones: present (normal negative)**

Protein: negative (normal negative)

Blood: negative (normal <3 red blood cells)

Bilirubin: negative (normal negative)

Leukocyte esterase: negative (normal negative)

Nitrite: negative (normal negative)

Bacteria: negative (normal negative)

**7. Urine and Serum Toxicity Studies**

Urine drug screen: negative

Serum acetaminophen: <10 mcg/mL (normal <10 mcg/mL)

Serum alcohol: <10 mg/dL (normal <10 mg/dL)

**Serum salicylate: 80 mg/dL (<5 normal range)**

**8. Serum osmolarity:**

Osmolarity: 290 (278-300 mmol/L)

**9. Lactic acid:**

**Lactic acid: 3.9 mmol/L (0.5-2.2 normal range)**
