# Supplementary material for: A Simulated Case of Acute Salicylate Toxicity From an Intentional Overdose
Source: MedEdPORTAL. 2018 Feb 12;14:10678. doi: 10.15766/mep_2374-8265.10678 (PMC6342373; doi:10.15766/mep_2374-8265.10678)
Supplement: Supplementary file 1 — A. Simulation Case.docx B. Actor Scripts.docx C. Preparation Assignment.docx D. Introduction to Activity.docx E. Lab and Diagnostic Results.docx F. Treatment Options.docx G. Survey Instrument.docx H. Debriefing Questions and Answers.docx I. Debriefing Session PowerPoint.pptx J. Abbreviated Debriefing Questions and Answers.docx [file mep-14-10678-s001.zip › F._Treatment_Options.docx]

**Appendix F: Treatment Options**

**1. Activated Charcoal**


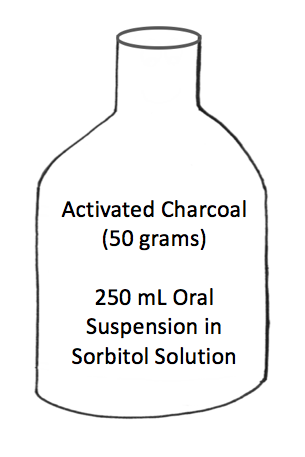


*Image created by Maria Sheakley, PhD (author)*

**2. Whole Bowel Irrigation**

Oral administration of an osmotic laxative with polyethylene glycol,

(e.g., Miralax)


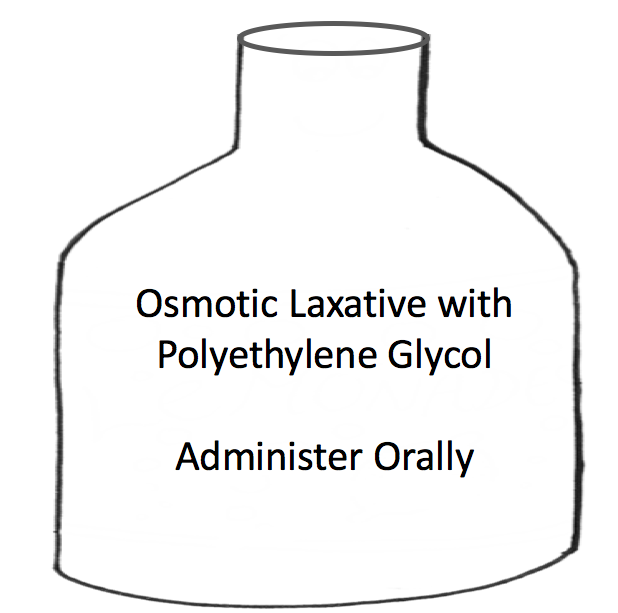


*Image created by Maria Sheakley, PhD (author)*
