# Supplementary material for: A Simulated Case of Acute Salicylate Toxicity From an Intentional Overdose
Source: MedEdPORTAL. 2018 Feb 12;14:10678. doi: 10.15766/mep_2374-8265.10678 (PMC6342373; doi:10.15766/mep_2374-8265.10678)
Supplement: Supplementary file 1 — A. Simulation Case.docx B. Actor Scripts.docx C. Preparation Assignment.docx D. Introduction to Activity.docx E. Lab and Diagnostic Results.docx F. Treatment Options.docx G. Survey Instrument.docx H. Debriefing Questions and Answers.docx I. Debriefing Session PowerPoint.pptx J. Abbreviated Debriefing Questions and Answers.docx [file mep-14-10678-s001.zip › G._Survey_Instrument.docx]

**Appendix G – Survey instrument**

| **Instructions:** rate how strongly you agree or disagree with each of the following statements about the Salicylate Toxicity Simulation event by circling the appropriate box, and provide written comments if desired. | | | | | | |
| --- | --- | --- | --- | --- | --- | --- |
|  | | Strongly Disagree | Disagree | Neutral | Agree | Strongly Agree |
| 1 | Pre-reading assignments prepared me for the salicylate toxicity simulation activity. | 1 | 2 | 3 | 4 | 5 |
| 2 | Briefing before the simulation was beneficial | 1 | 2 | 3 | 4 | 5 |
| 3 | Briefing before the simulation increased my confidence. | 1 | 2 | 3 | 4 | 5 |
| 4 | During the simulation, I had the opportunity to practice my clinical decision-making skills. | 1 | 2 | 3 | 4 | 5 |
| 5 | During the simulation, I had the opportunity to experience how time pressure can affect my clinical decision-making skills. | 1 | 2 | 3 | 4 | 5 |
| 6 | During the simulation, I had the opportunity to work as part of a healthcare team. | 1 | 2 | 3 | 4 | 5 |
| 7 | I am more confident in my ability to report information to my health care team. | 1 | 2 | 3 | 4 | 5 |
| 8 | I am more confident in my understanding of the pathophysiology of salicylate toxicity | 1 | 2 | 3 | 4 | 5 |
| 9 | I am more confident in my ability to differentiate between different types acid-base disturbances | 1 | 2 | 3 | 4 | 5 |
| 10 | Debriefing contributed to my learning. | 1 | 2 | 3 | 4 | 5 |
| 11 | Debriefing was valuable in helping me select the appropriate treatments for salicylate toxicity | 1 | 2 | 3 | 4 | 5 |
| 12 | Debriefing provided adequate time to review the critical concepts related to salicylate toxicity, including acid-base disturbances | 1 | 2 | 3 | 4 | 5 |
| 13 | Debriefing provided opportunities to self-reflect on my performance during the simulation. | 1 | 2 | 3 | 4 | 5 |
| Comments (optional): | | | | | | |
