# Supplementary material for: A Simulated Case of Acute Salicylate Toxicity From an Intentional Overdose
Source: MedEdPORTAL. 2018 Feb 12;14:10678. doi: 10.15766/mep_2374-8265.10678 (PMC6342373; doi:10.15766/mep_2374-8265.10678)
Supplement: Supplementary file 1 — A. Simulation Case.docx B. Actor Scripts.docx C. Preparation Assignment.docx D. Introduction to Activity.docx E. Lab and Diagnostic Results.docx F. Treatment Options.docx G. Survey Instrument.docx H. Debriefing Questions and Answers.docx I. Debriefing Session PowerPoint.pptx J. Abbreviated Debriefing Questions and Answers.docx [file mep-14-10678-s001.zip › H._Debriefing_Questions_and_Answers.docx]

**Appendix H: Debriefing Questions and Answers**

**SALICYLATE OVERDOSE CASE SIMULATION**

**1. Who assumed the role of scribe(s) for your group(s)?**

One of the scribes will present the patient’s clinical findings.

**2. Present the history and physical exam that you obtained from this patient. Include all abnormal findings and pertinent negative findings.** (10 mins)

**A. List the essential general information**

Age: 23 years

Male (or female)

Generally healthy

History of depression

Currently suicidal

Took an overdose of aspirin (non-enteric coated)

Time of ingestion was 3 hours ago

Number of pills taken = 40

Patient’s weight = 70 kg

Not taking (other) medications, recreational drugs, or alcohol

**B. List the essential symptoms**

Nausea

Abdominal pain

Diaphoresis

Tachypnea

Tinnitus

**C. List the essential physical findings**

Blood Pressure: 110/76

Pulse: 118 beats per minute

Respiratory rate: 28 breaths per minute

Temp: 38.0 degrees C (100.4 degrees F)

**D. List the abnormal physical findings that you observed in this patient, from head-to-toe: (normal findings are in light gray text).**

General appearance: Diaphoretic (sweaty), tachypneic (fast breathing), repeated emesis (vomiting), streaks of blood are visible in the emesis

Head: Normal

Eyes: Pupils equal to light and accommodation

Normal conjunctiva

Ears: Normal tympanic membrane without effusion; patient is hard of hearing

Nose: Normal

Mouth & Throat: Dry mucus membranes

Neck: Normal

Chest: Normal

Lungs (auscultation): Breath sounds equal on both sides; Clear to auscultation bilaterally; tachypneic

Heart (auscultation): Tachycardia (fast heart rate)

Regular heart rate (no skipped beats)

Abdomen/rectal: Normoactive bowel sounds on auscultation; diffusely tender to palpation (touch)

Genitalia: Normal male genitalia

Extremities/Musculoskeletal: Normal muscle tone

Vascular: Strong pulses in all extremities

Skin: Profuse diaphoresis (sweating); Normal color; Warm to touch

Mental status: Alert and oriented; mild restlessness, mild agitation

Neurologic: Motor function intact (moves all 4 extremities with

normal strength)

**3. How do you explain each of these physical findings on the basis of alterations in physiology caused by this drug?**

Elevated temperature (hyperpyrexia): Salicylates uncouple oxidative phosphorylation in the mitochondria; this generates heat.

Tachycardia: due to low BP caused by vasodilation, low blood volume from emesis and diaphoresis, and to a lesser extent, fever and acidosis.

Tachypnea: Direct stimulation of medullary respiratory center by salicylate, attempting to compensate for metabolic acidosis

Diaphoresis: Hypothalamic induction of peripheral vasodilation for antipyresis

Dry mouth: Dehydration from insensible losses (lungs, fever), vomiting, osmotic diuresis, and tachypnea.

Abdominal pain & emesis (with blood): Irritation of the gastric mucosa; inhibition of cyclooxygenase, direct stimulation of medullary chemoreceptors

Restlessness & agitation: Direct toxicity of salicylate in the central nervous system (CNS), neuroglycopenia (low glucose in the cerebral spinal fluid and brain), and cerebral edema (swelling of the brain). Salicylate toxicity increases CNS utilization of glucose, and serum glucose levels may not reflect CNS levels.

Tinnitus: Direct cochlear effect and cerebral effect

**4. Laboratory data, with normal ranges, are as follows:**

**Comprehensive Metabolic Panel (CMP)**

Na: (sodium) 141 mEq/L (135-144 normal range)

K (potassium): 3.8 mEq/L (3.7-5.2 normal range)

Cl (chloride): 101 mmol/L (101-111 normal range)

**CO2 (bicarbonate): 12 mmol/L (20-29 normal range; use 25 as normal)**

BUN (blood urea nitrogen): **25 mg/dL** (7-20 normal range)

Cr (creatinine): 1.3 mg/dL (0.8-1.4 normal range)

Glucose: 85 mg/dL (64-128 normal range)

AST (aspartate aminotransferase): 40 U/L (8-48 normal range)

ALT (alanine aminotransferase): 42 U/L (7-55 normal range)

Alkaline phosphatase: 99 U/L (45-115 normal range)

Total bilirubin: 0.8 mg/dL (0.1-1 normal range)

Albumin: 4 g/dL (3.5-5 normal range)

**Complete Blood Count (CBC) without differential**

White blood cells: 9.2 billion cells/L (3.5-10.5 normal range)

Hemoglobin: 14.0 g/dL (13.5-17.5 normal range)

Hematocrit: 42% (38.8-50%)

Platelets: 350 billion/L (150-450 normal range)

**Arterial Blood Gas (ABG) on room air**

**pH: 7.31 (7.38-7.42 normal range)**

**PCO2: 22 mm Hg (38-42 normal range)**

PO2: 95 mm Hg (80-100 normal range)

**Urinalysis**

Specific gravity: 1.010 (1.005-1.025 normal range)

Color: yellow

pH: 5.5 (4.5-8 normal range)

**Ketones: present (normal negative)**

Protein: negative (normal negative)

Blood: negative (normal <3 red blood cells)

Bilirubin: negative (normal negative)

Leukocyte esterase: negative (normal negative)

Nitrite: negative (normal negative)

Bacteria: negative (normal negative)

Drug screen: negative

Serum acetaminophen: <10 mcg/mL (normal <10 mcg/mL)

Serum alcohol: <10 mg/dL (normal <10 mg/dL)

**Serum salicylate: 80 mg/dL (<5 normal range)**

Serum osmolality: 290 (278-300 mmol/L)

**Lactic acid: 3.9 mmol/L (0.5-2.2 normal range)**

**5. Why is the arterial pCO_2_ decreased in salicylate toxicity?**

The reasons for decreased pCO_2_ are:

(1) Stimulation of the respiratory center causes tachypnea; more carbon dioxide is exhaled; increased respiratory rate drives down serum CO_2_. This causes a respiratory alkalosis.

(2) Hyperventilation is a compensatory mechanism for a metabolic acidosis.


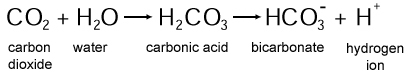


**6. What are the four main types of acid-base disturbances?**

Respiratory acidosis (eg. inadequate ventilations)

Respiratory alkalosis (eg. hyperventilation)

Metabolic acidosis

Metabolic alkalosis

**7. What organ systems compensate for the metabolic effects of salicylates?**

Lungs

Kidneys

**8. What type(s) of acid-base disturbance(s) did this patient have at the time of presentation?**

Respiratory alkalosis (primary process, then compensatory)

Metabolic acidosis (primary process)

Anion gap

**9. Did the patient ingest a potentially toxic dose of salicylate?**

The 70-kg patient ingested ~200 mg/kg of salicylate. (Forty pills x 325 mg = 13 grams)

In a range of 150-300 mg/kg, moderate toxicity is expected. (300 mg/kg is severely toxic, and < 150 mg/kg is minimally toxic.

Physical findings and the time to peak plasma concentrations will be delayed if the patient ingested an enteric-coated aspirin, (which was not the case with this patient). Absorption may be variable. To insure that the peak level has been reached (and passed), obtain serial levels until at least two levels are found to be declining.

**10. How toxic was the patient (mild/moderate/severe), and how did you decide?**

A serum salicylate level is 80 mg/dL. The therapeutic range is 10-30 mg/dL; early signs of toxicity are seen at 40-50 mg/dL; and >100 mg/dL (or serious signs) is an indication for hemodialysis. Serious toxicity can occur at levels as low as 60-80 mg/d, so combine serum level and clinical findings to determine the severity of the poisoning.

Symptoms suggest moderate toxicity.

**11. What are the four main methods of treatment for salicylate poisoning, and why are they done?**

1. IV fluids—for rehydration
2. GI decontamination—to prevent further absorption through charcoal-binding
3. Sodium bicarbonate—to promote excretion of the salicylate
4. Hemodialysis—for severe poisoning, to eliminate the poisoning from the bloodstream

GI decontamination is a controversial issue in Toxicology. There are three primary methods: gastric emptying, prevention of absorption with activated charcoal, and enhanced elimination with a process called “whole bowel irrigation”. All of these methods can be used, but for very specific indications. The decision to empty the stomach with an “orogastric hose” is complicated, and it is nicely described in a widely used toxicology textbook if you are interested. (Reference: Hoegberg LCG, Gude AB: Techniques used to prevent gastrointestinal absorption: in Hoffman RS, Howland MA, Lewin NA, Nelson LS, Goldfrank LR (eds), Goldfrank’s Toxicologic Emergencies; 10th ed. New York, McGraw-Hill Education; 2015: 83-96. See Tables 8-1 and 8-2.) Activated charcoal will bind poisons that are in the stomach and reduce systemic absorption if the ingestion occurred recently or if gastric emptying is delayed. It can even interrupt the enterohepatic circulation of some substances. Whole bowel irrigation using an osmotically balanced polyethylene glycol lavage solution essentially “purges” the GI tract of the poison. Activated charcoal and whole bowel irrigation are reasonable treatment options for this patient. The ingestion occurred too long ago for gastric emptying to be effective with this particular substance.

**12. You chose to treat (or could have treated) this patient with sodium bicarbonate. How would it benefit the patient? How would this drug affect acid/base balance? How would it manifest clinically?**

- Treatment with sodium bicarbonate increases the serum pH.
- Increase in pH (alkalinization) shifts the equilibrium of salicylate to the ionized state.
- In the ionized state, salicylates are prevented from entering other organs (most importantly the brain).
- Called “ion trapping” because ionized salicylate is trapped in the plasma, and thus cannot pass into tissues.
- Serum alkalinization results in urine alkalinization.
- Traps ionized salicylate in the renal tubules and enhances excretion in the urine.
- If serum potassium is low, repletion is needed for sodium bicarbonate to work. (Renal hydrogen-potassium pump)

**13. These are the indications for hemodialysis in the setting of acute salicylate toxicity. (List on a slide.) Did your patient have any of them?**

1. Altered mental status/cerebral edema *(confusion, somnolence)* **Marginal**
2. Pulmonary edema *(noisy lung sounds)* **No**
3. Renal insufficiency that interferes with salicylate excretion *(no urine output)* **No**
4. Fluid overload that prevents the administration of sodium bicarbonate *(noisy lung sounds)* **No**
5. A serum salicylate concentration >100 mg/dL in acute overdose **No**
6. Clinical deterioration despite aggressive and appropriate supportive care *(over time)* **No**
