# Supplementary material for: A Simulated Case of Acute Salicylate Toxicity From an Intentional Overdose
Source: MedEdPORTAL. 2018 Feb 12;14:10678. doi: 10.15766/mep_2374-8265.10678 (PMC6342373; doi:10.15766/mep_2374-8265.10678)
Supplement: Supplementary file 1 — A. Simulation Case.docx B. Actor Scripts.docx C. Preparation Assignment.docx D. Introduction to Activity.docx E. Lab and Diagnostic Results.docx F. Treatment Options.docx G. Survey Instrument.docx H. Debriefing Questions and Answers.docx I. Debriefing Session PowerPoint.pptx J. Abbreviated Debriefing Questions and Answers.docx [file mep-14-10678-s001.zip › I._Debriefing_Session_PowerPoint.pptx]

## Slide 1
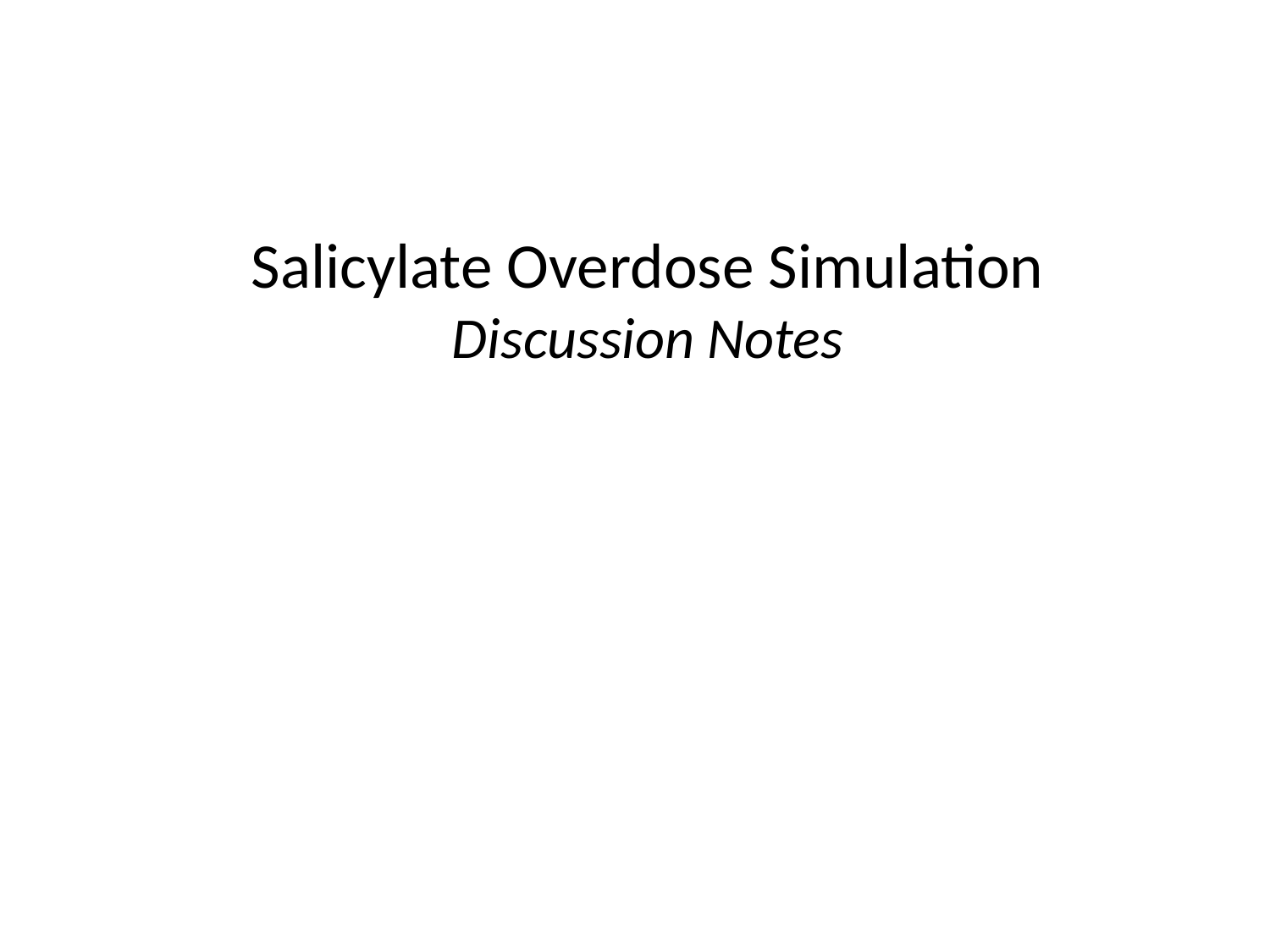

# Salicylate Overdose Simulation Discussion Notes

## Slide 2
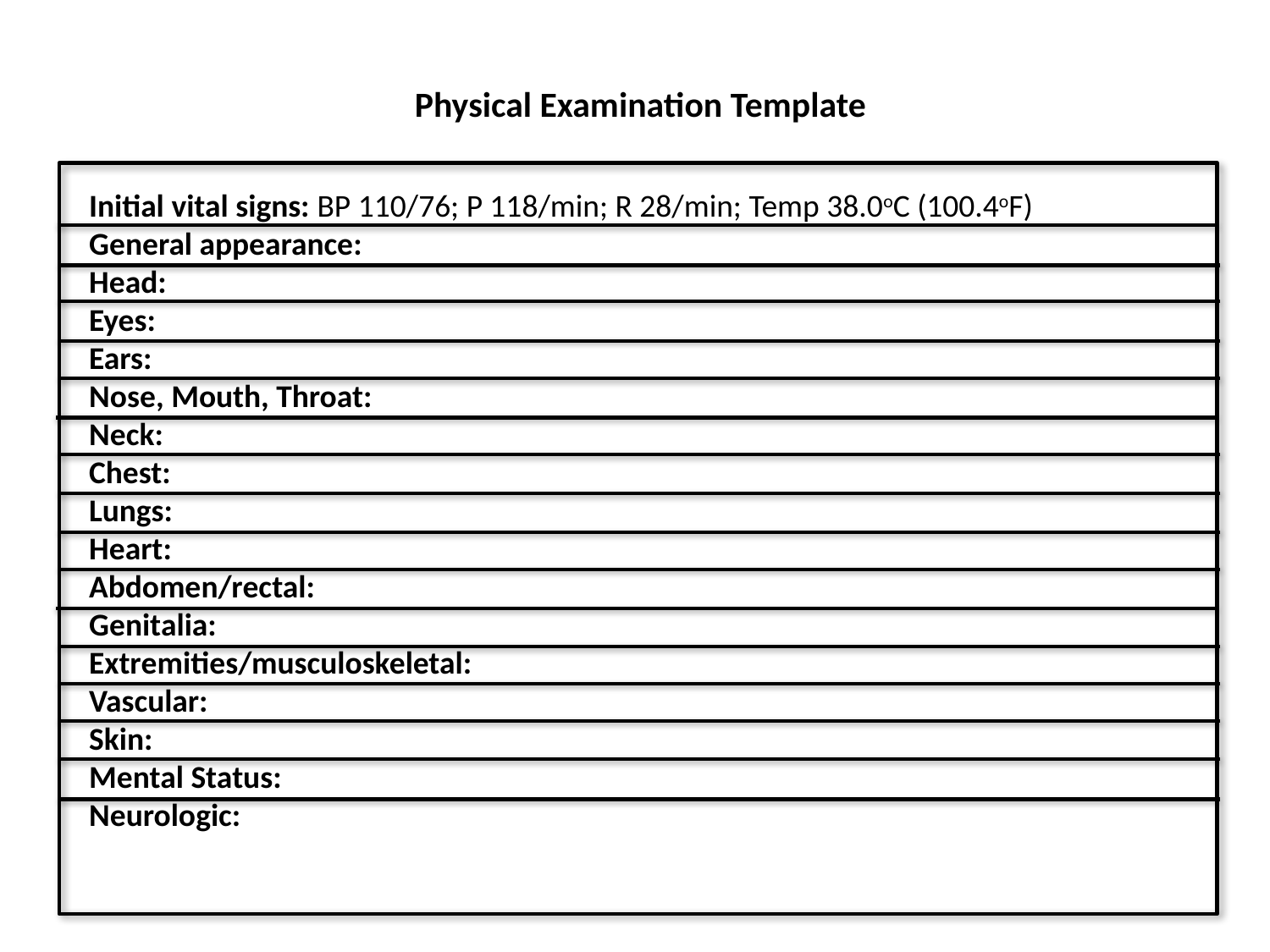

Physical Examination Template
Initial vital signs: BP 110/76; P 118/min; R 28/min; Temp 38.0oC (100.4oF)
General appearance:
Head:
Eyes:
Ears:
Nose, Mouth, Throat:
Neck:
Chest:
Lungs:
Heart:
Abdomen/rectal:
Genitalia:
Extremities/musculoskeletal:
Vascular:
Skin:
Mental Status:
Neurologic:

## Slide 3
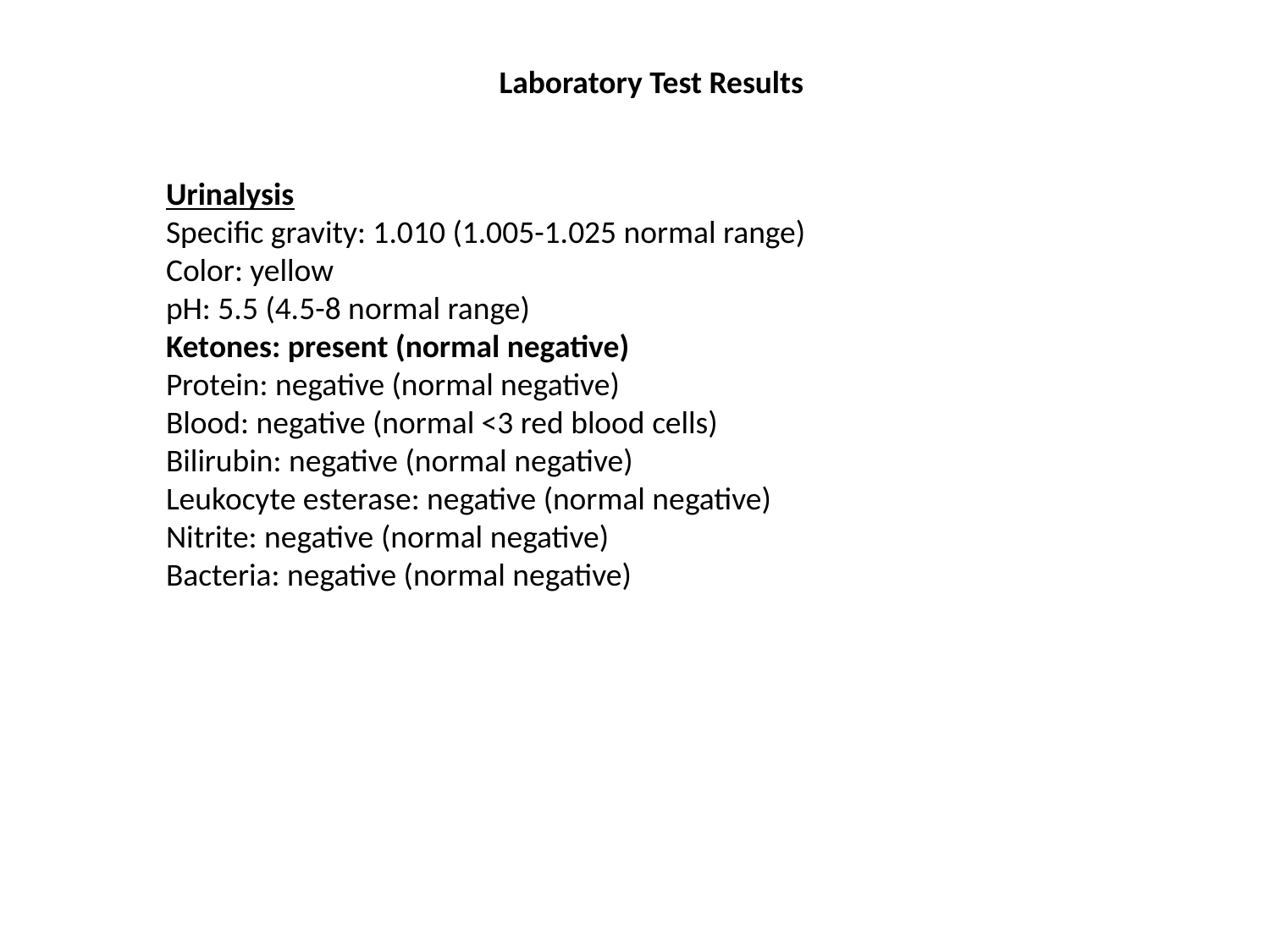

Laboratory Test Results
Urinalysis
Specific gravity: 1.010 (1.005-1.025 normal range)
Color: yellow
pH: 5.5 (4.5-8 normal range)
Ketones: present (normal negative)
Protein: negative (normal negative)
Blood: negative (normal <3 red blood cells)
Bilirubin: negative (normal negative)
Leukocyte esterase: negative (normal negative)
Nitrite: negative (normal negative)
Bacteria: negative (normal negative)

## Slide 4
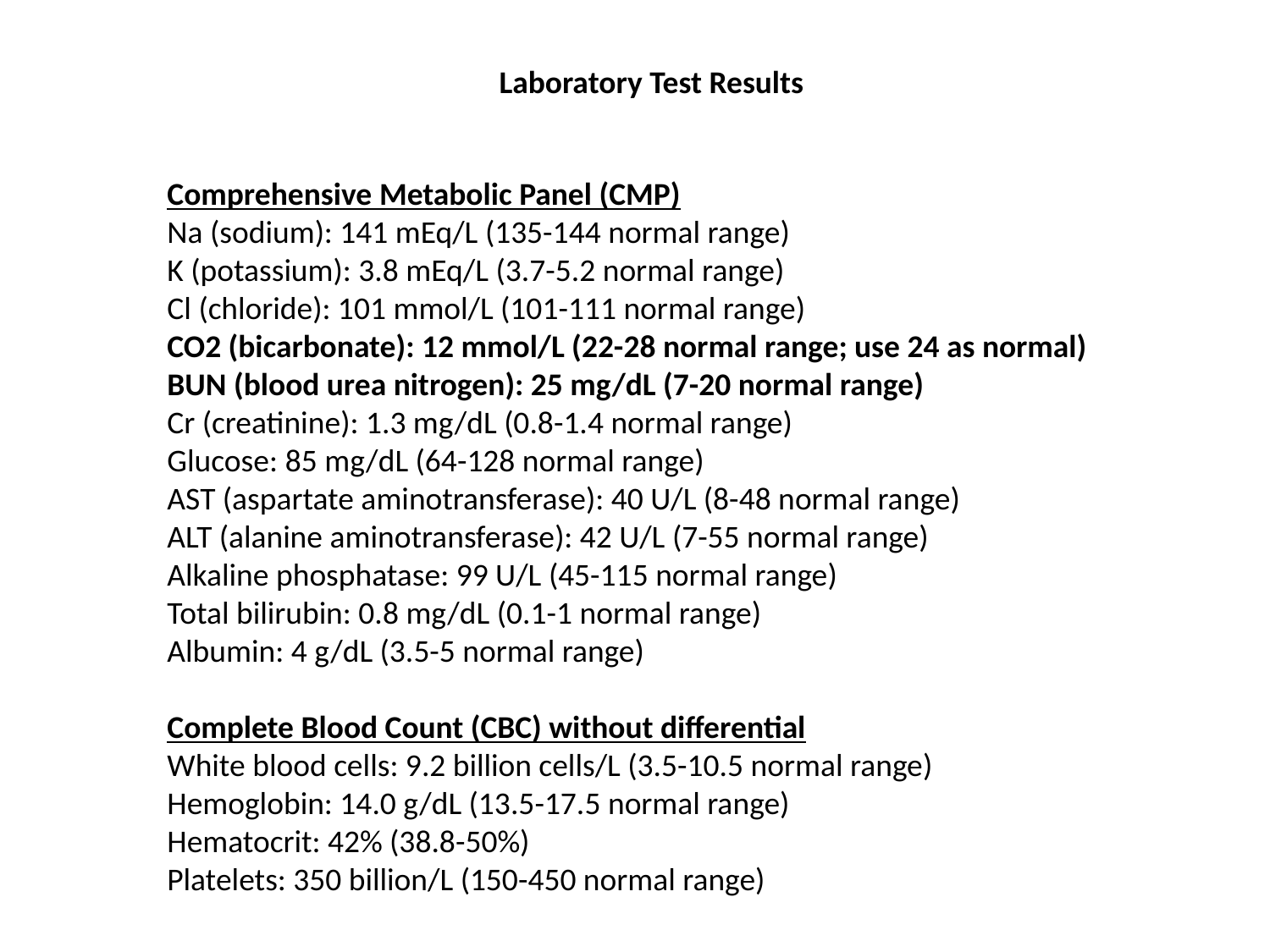

Laboratory Test Results
Comprehensive Metabolic Panel (CMP)
Na (sodium): 141 mEq/L (135-144 normal range)
K (potassium): 3.8 mEq/L (3.7-5.2 normal range)
Cl (chloride): 101 mmol/L (101-111 normal range)
CO2 (bicarbonate): 12 mmol/L (22-28 normal range; use 24 as normal)
BUN (blood urea nitrogen): 25 mg/dL (7-20 normal range)
Cr (creatinine): 1.3 mg/dL (0.8-1.4 normal range)
Glucose: 85 mg/dL (64-128 normal range)
AST (aspartate aminotransferase): 40 U/L (8-48 normal range)
ALT (alanine aminotransferase): 42 U/L (7-55 normal range)
Alkaline phosphatase: 99 U/L (45-115 normal range)
Total bilirubin: 0.8 mg/dL (0.1-1 normal range)
Albumin: 4 g/dL (3.5-5 normal range)
Complete Blood Count (CBC) without differential
White blood cells: 9.2 billion cells/L (3.5-10.5 normal range)
Hemoglobin: 14.0 g/dL (13.5-17.5 normal range)
Hematocrit: 42% (38.8-50%)
Platelets: 350 billion/L (150-450 normal range)

## Slide 5
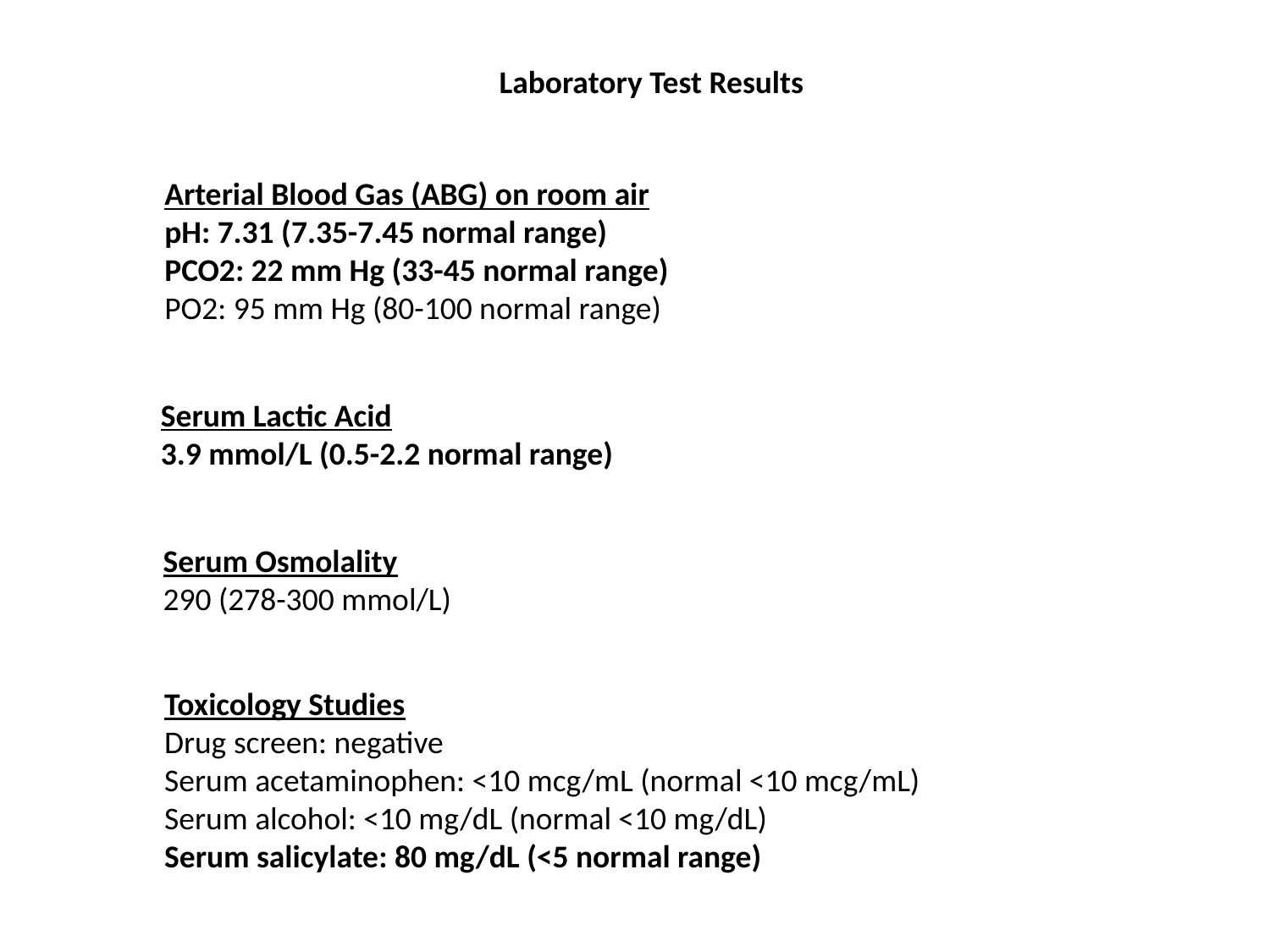

Laboratory Test Results
Arterial Blood Gas (ABG) on room air
pH: 7.31 (7.35-7.45 normal range)
PCO2: 22 mm Hg (33-45 normal range)
PO2: 95 mm Hg (80-100 normal range)
Serum Lactic Acid
3.9 mmol/L (0.5-2.2 normal range)
Serum Osmolality
290 (278-300 mmol/L)
Toxicology Studies
Drug screen: negative
Serum acetaminophen: <10 mcg/mL (normal <10 mcg/mL)
Serum alcohol: <10 mg/dL (normal <10 mg/dL)
Serum salicylate: 80 mg/dL (<5 normal range)

## Slide 6
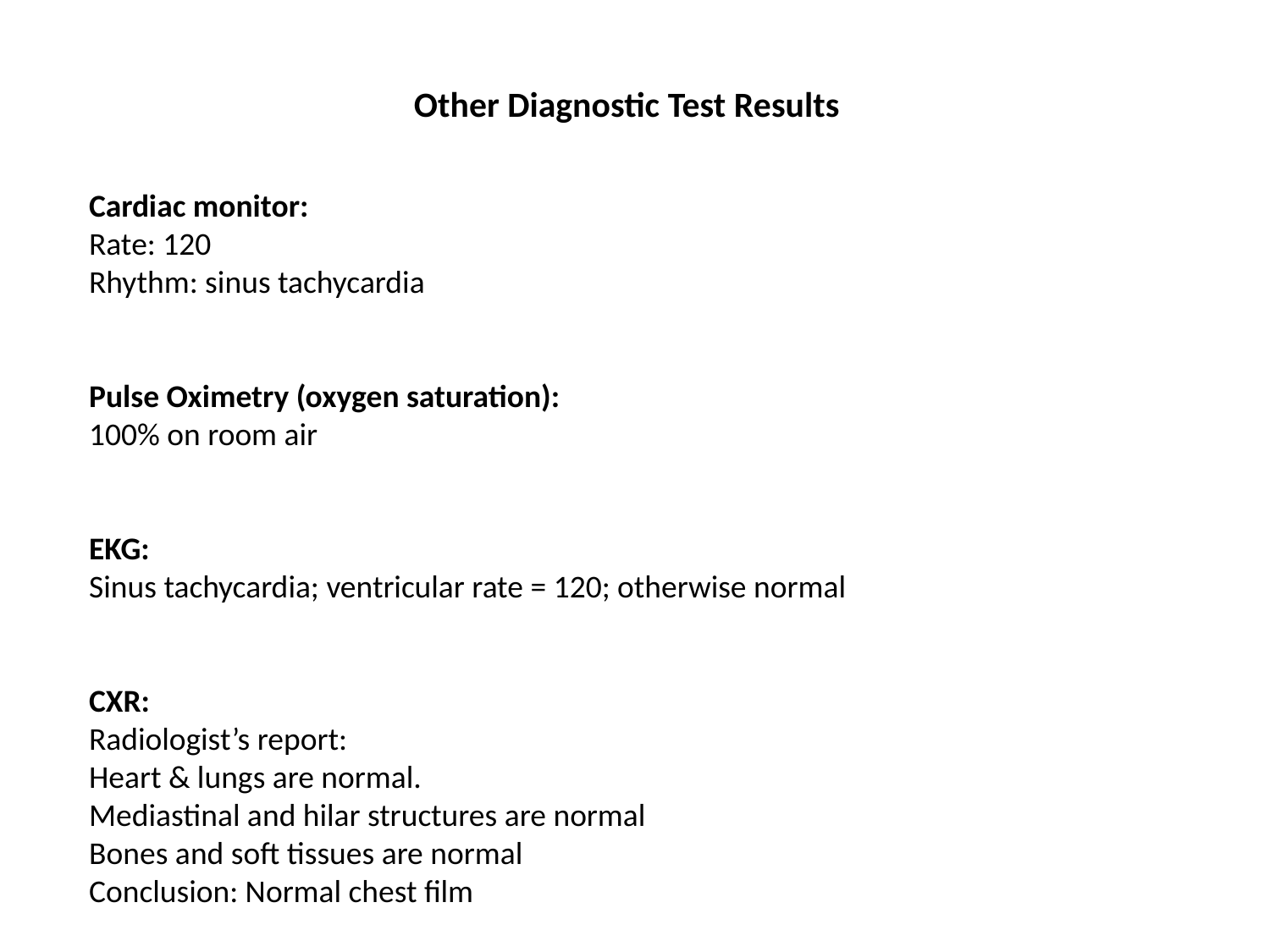

Other Diagnostic Test Results
Cardiac monitor:
Rate: 120
Rhythm: sinus tachycardia
Pulse Oximetry (oxygen saturation):
100% on room air
EKG:
Sinus tachycardia; ventricular rate = 120; otherwise normal
CXR:
Radiologist’s report:
Heart & lungs are normal.
Mediastinal and hilar structures are normal
Bones and soft tissues are normal
Conclusion: Normal chest film

## Slide 7
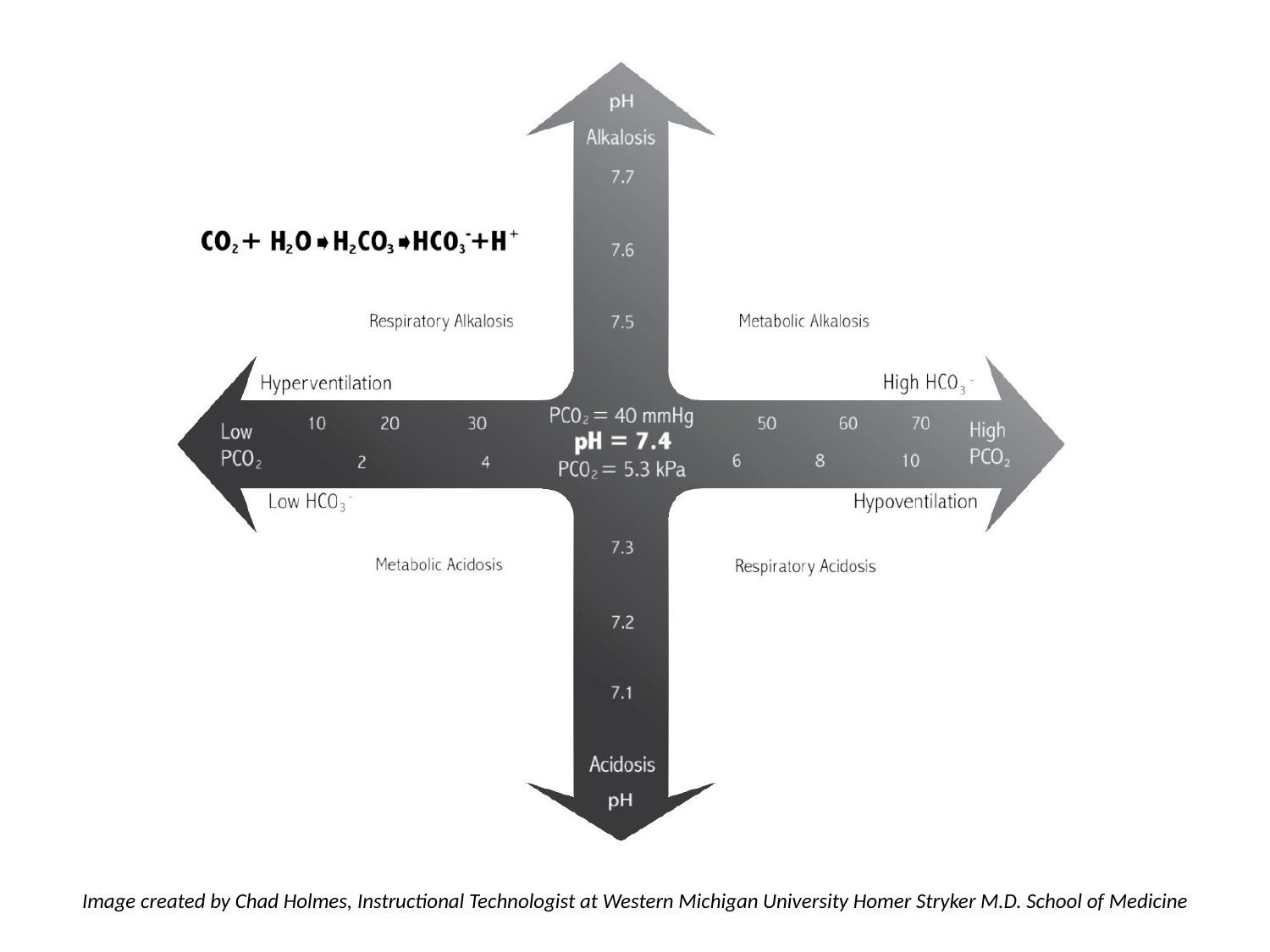

Image created by Chad Holmes, Instructional Technologist at Western Michigan University Homer Stryker M.D. School of Medicine

## Slide 8
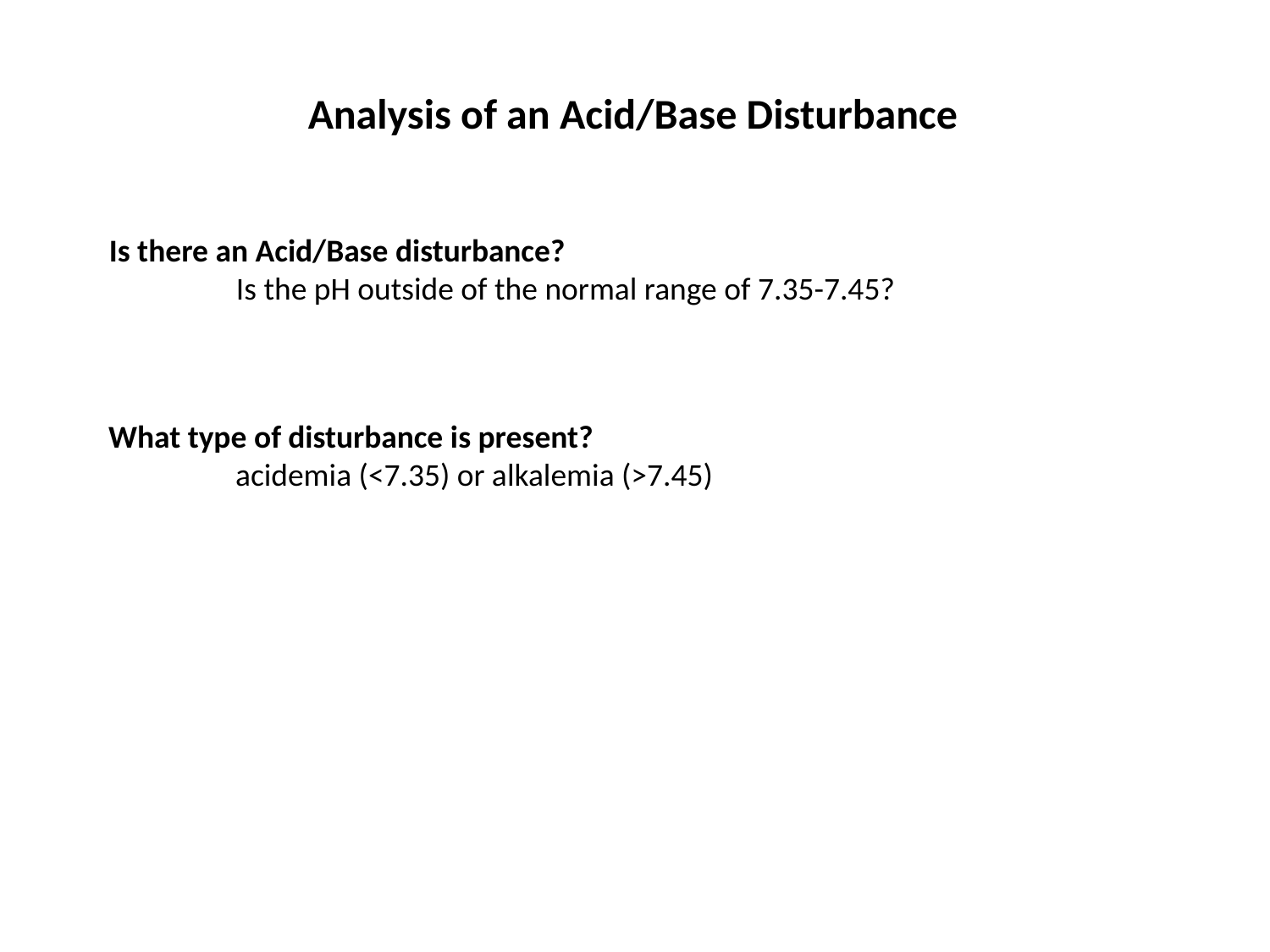

Analysis of an Acid/Base Disturbance
Is there an Acid/Base disturbance?
	Is the pH outside of the normal range of 7.35-7.45?
What type of disturbance is present?
	acidemia (<7.35) or alkalemia (>7.45)

## Slide 9
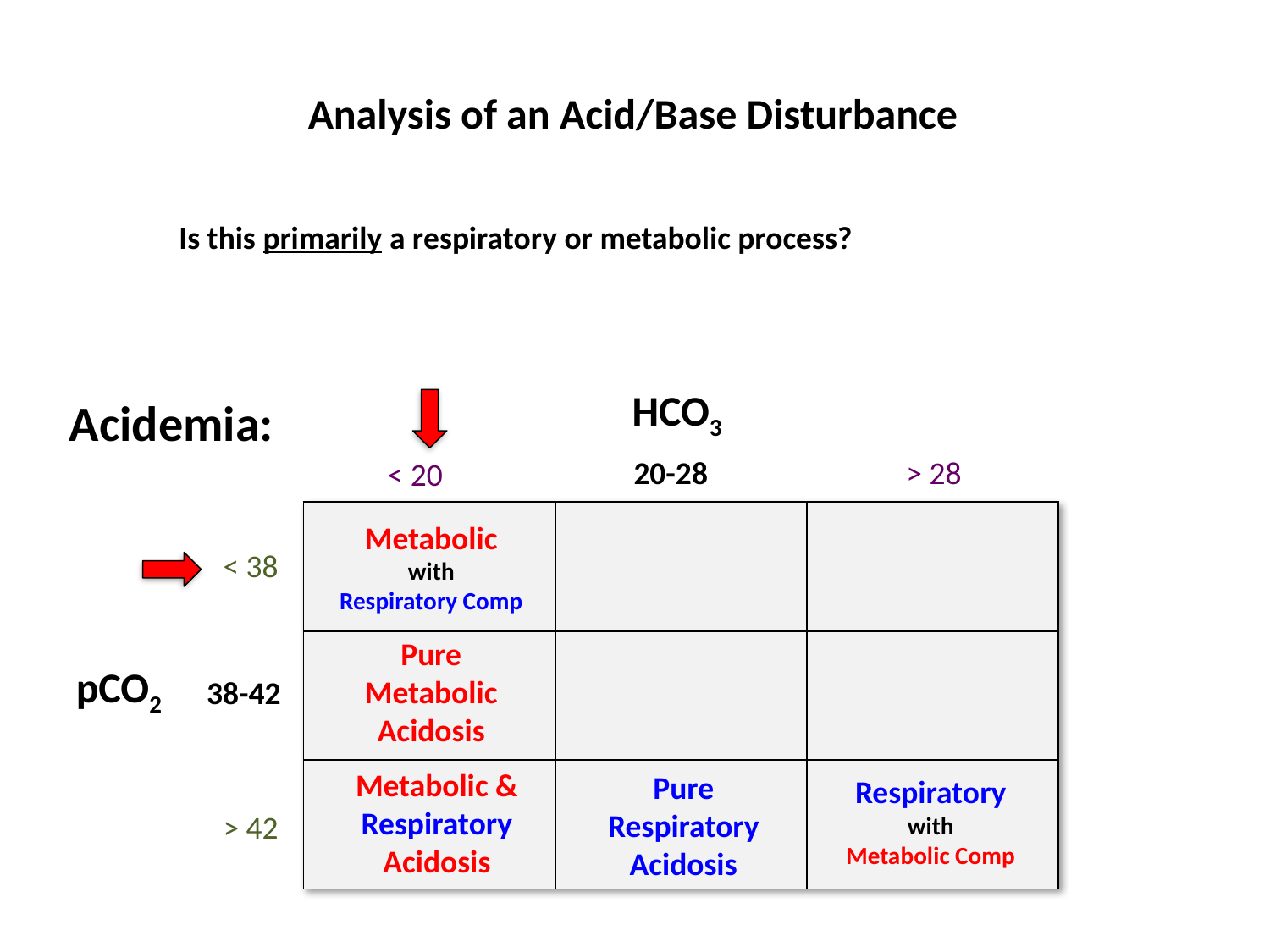

Analysis of an Acid/Base Disturbance
Is this primarily a respiratory or metabolic process?
HCO3
Acidemia:
20-28
> 28
< 20
| | | |
| --- | --- | --- |
| | | |
| | | |
Metabolic
with
Respiratory Comp
< 38
Pure
Metabolic
Acidosis
pCO2
38-42
Metabolic &
Respiratory
Acidosis
Pure
Respiratory
Acidosis
Respiratory
with
Metabolic Comp
> 42

## Slide 10
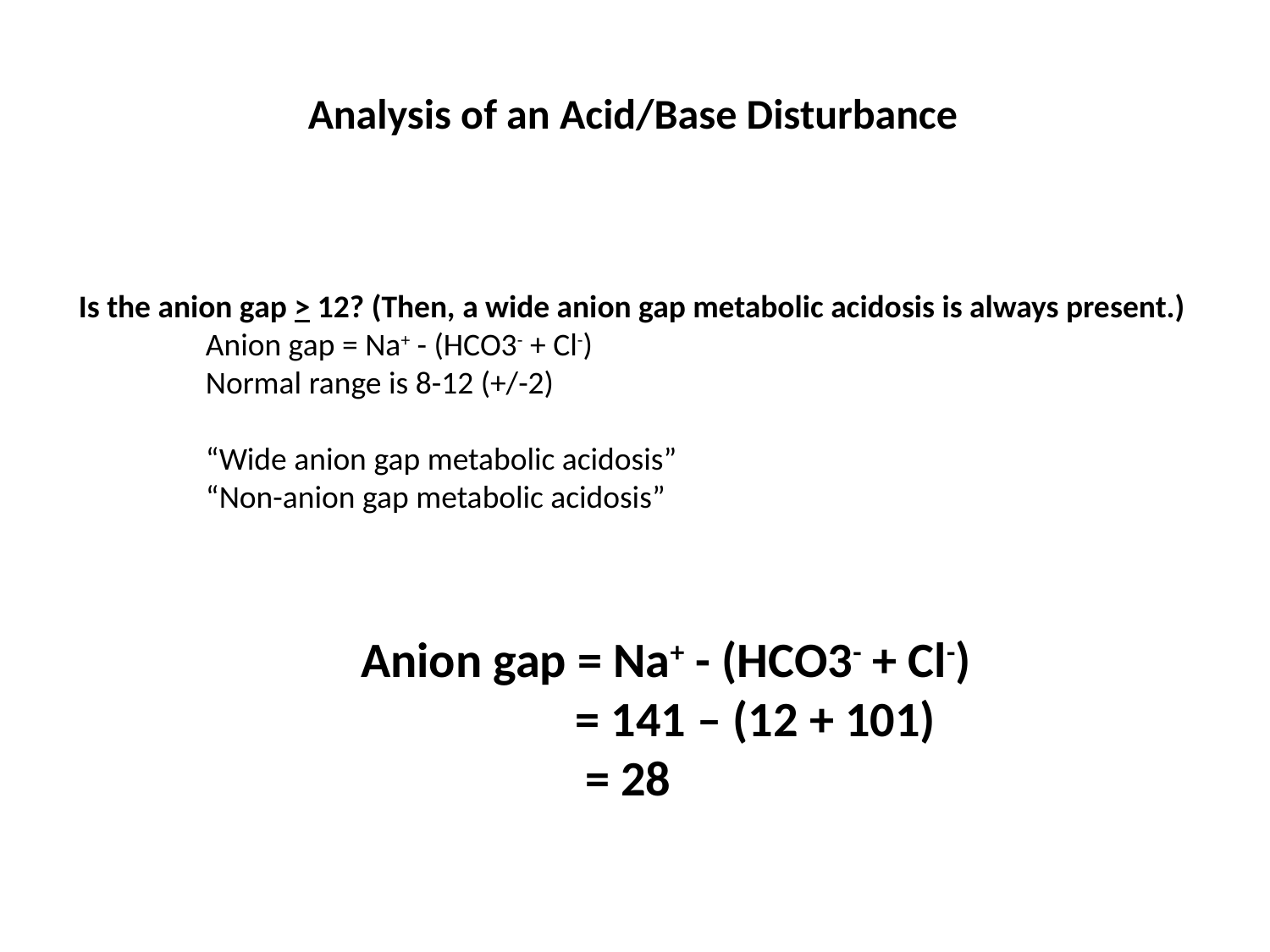

Analysis of an Acid/Base Disturbance
Is the anion gap > 12? (Then, a wide anion gap metabolic acidosis is always present.)
	Anion gap = Na+ - (HCO3- + Cl-)
	Normal range is 8-12 (+/-2)
	“Wide anion gap metabolic acidosis”
	“Non-anion gap metabolic acidosis”
Anion gap = Na+ - (HCO3- + Cl-)
 = 141 – (12 + 101)
 = 28

## Slide 11
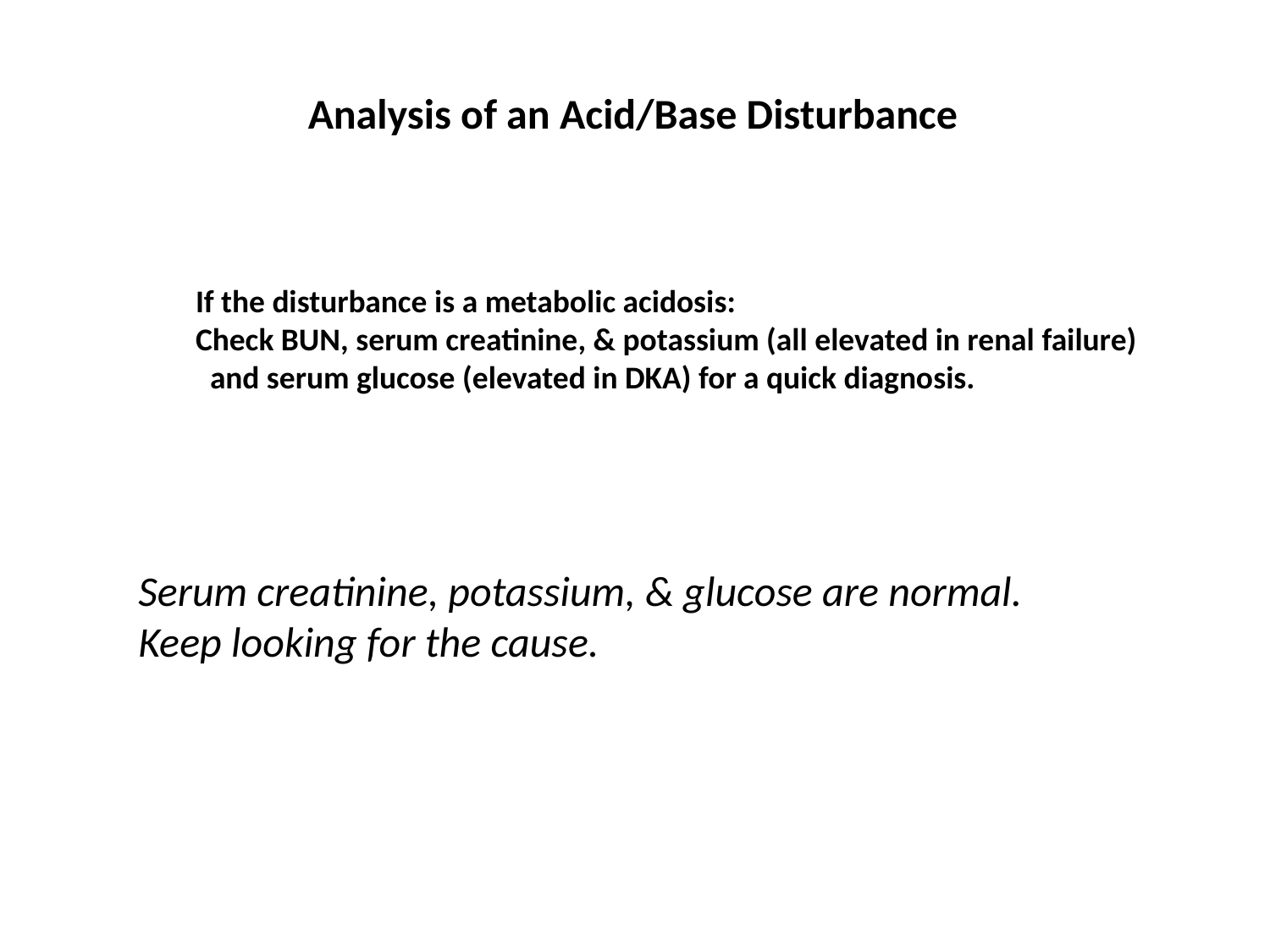

Analysis of an Acid/Base Disturbance
If the disturbance is a metabolic acidosis:
Check BUN, serum creatinine, & potassium (all elevated in renal failure)
 and serum glucose (elevated in DKA) for a quick diagnosis.
Serum creatinine, potassium, & glucose are normal.
Keep looking for the cause.

## Slide 12
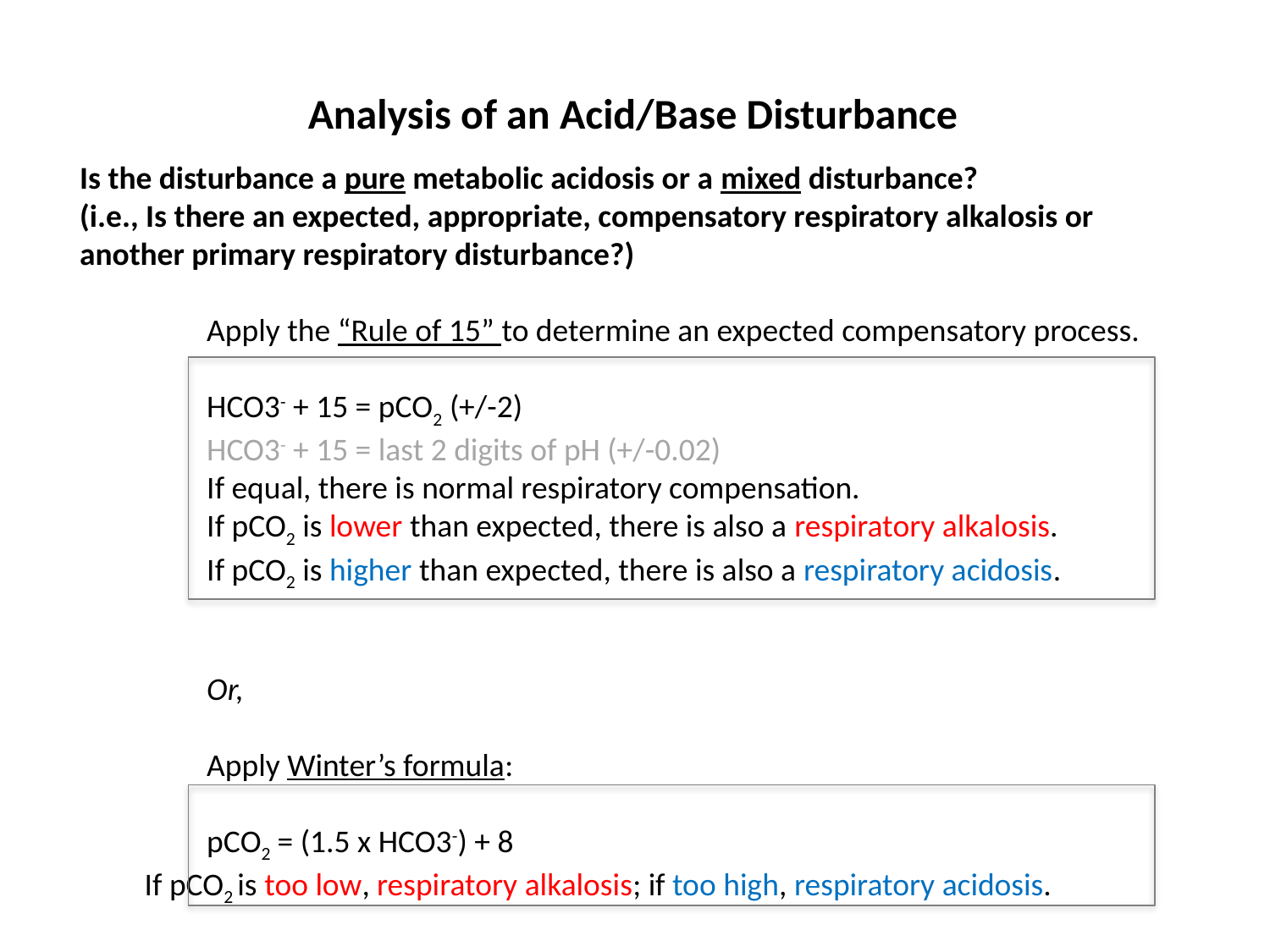

Analysis of an Acid/Base Disturbance
Is the disturbance a pure metabolic acidosis or a mixed disturbance?
(i.e., Is there an expected, appropriate, compensatory respiratory alkalosis or
another primary respiratory disturbance?)
	Apply the “Rule of 15” to determine an expected compensatory process.
	HCO3- + 15 = pCO2 (+/-2)
	HCO3- + 15 = last 2 digits of pH (+/-0.02)
	If equal, there is normal respiratory compensation.
	If pCO2 is lower than expected, there is also a respiratory alkalosis.
	If pCO2 is higher than expected, there is also a respiratory acidosis.
	Or,
	Apply Winter’s formula:
	pCO2 = (1.5 x HCO3-) + 8
 If pCO2 is too low, respiratory alkalosis; if too high, respiratory acidosis.

## Slide 13
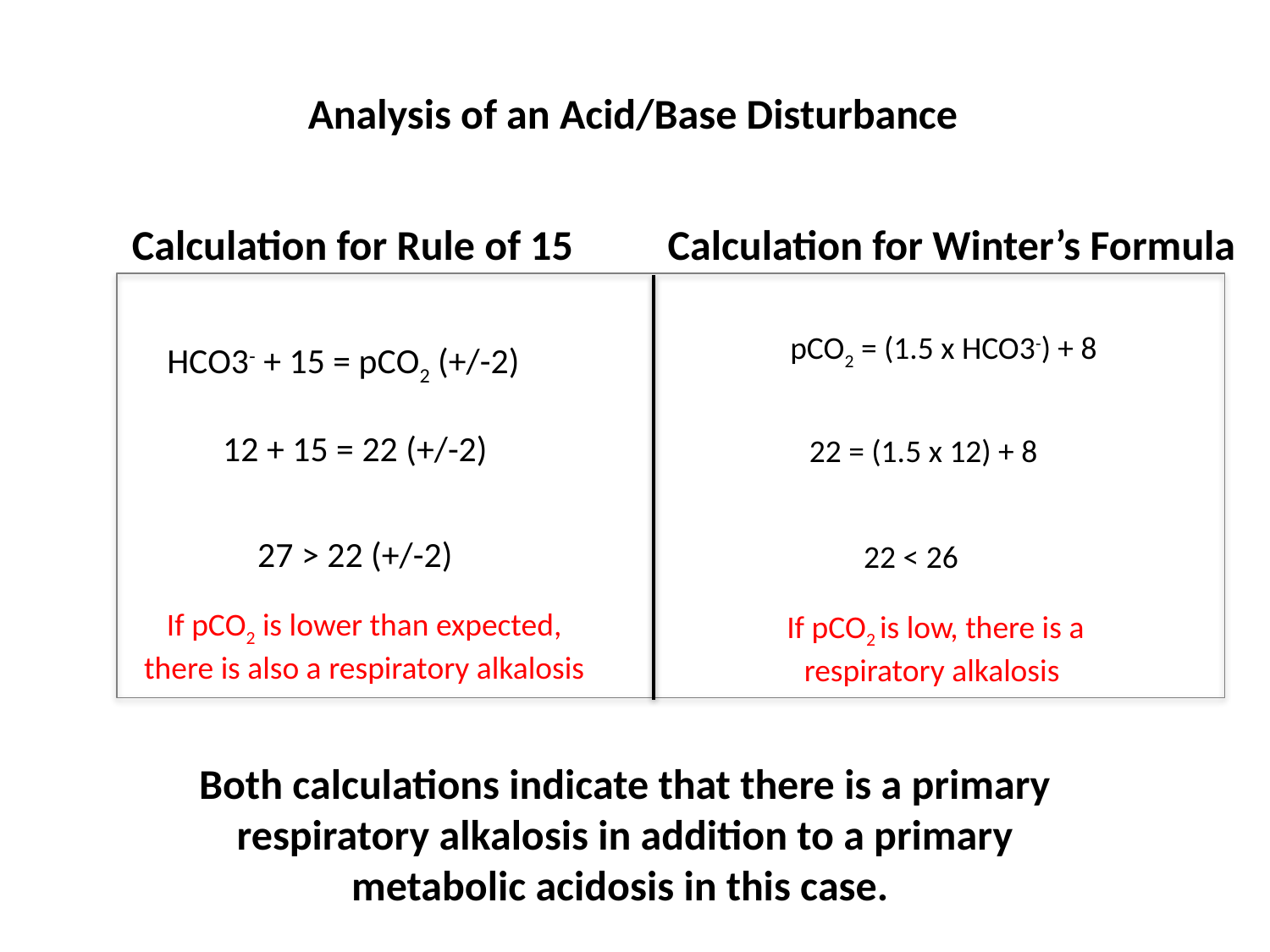

Analysis of an Acid/Base Disturbance
Calculation for Rule of 15
Calculation for Winter’s Formula
pCO2 = (1.5 x HCO3-) + 8
HCO3- + 15 = pCO2 (+/-2)
 12 + 15 = 22 (+/-2)
22 = (1.5 x 12) + 8
 27 > 22 (+/-2)
22 < 26
If pCO2 is lower than expected, there is also a respiratory alkalosis
 If pCO2 is low, there is a respiratory alkalosis
Both calculations indicate that there is a primary respiratory alkalosis in addition to a primary metabolic acidosis in this case.

## Slide 14
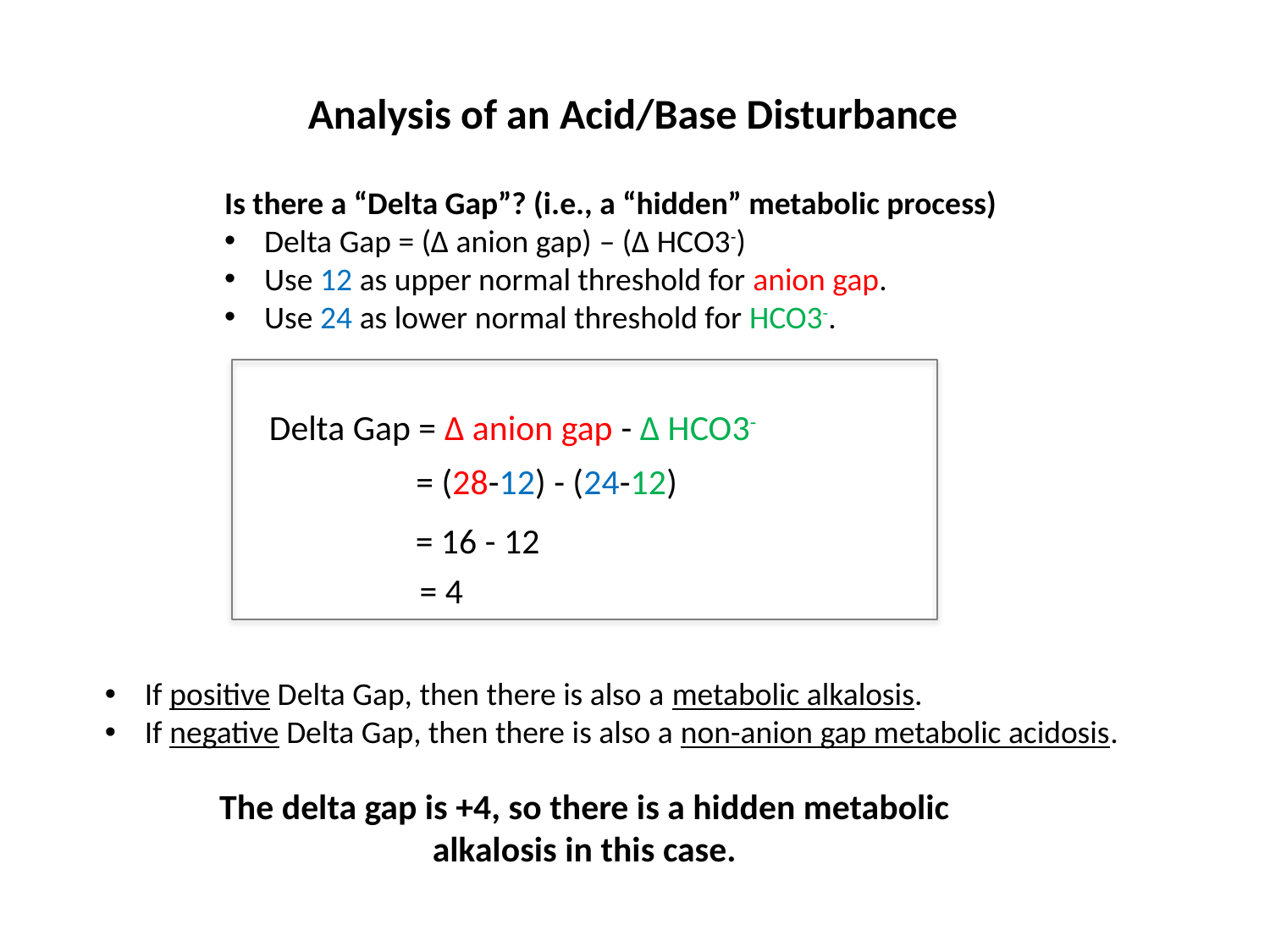

Analysis of an Acid/Base Disturbance
Is there a “Delta Gap”? (i.e., a “hidden” metabolic process)
Delta Gap = (Δ anion gap) – (Δ HCO3-)
Use 12 as upper normal threshold for anion gap.
Use 24 as lower normal threshold for HCO3-.
Delta Gap = Δ anion gap - Δ HCO3-
= (28-12) - (24-12)
= 16 - 12
= 4
If positive Delta Gap, then there is also a metabolic alkalosis.
If negative Delta Gap, then there is also a non-anion gap metabolic acidosis.
The delta gap is +4, so there is a hidden metabolic alkalosis in this case.

## Slide 15
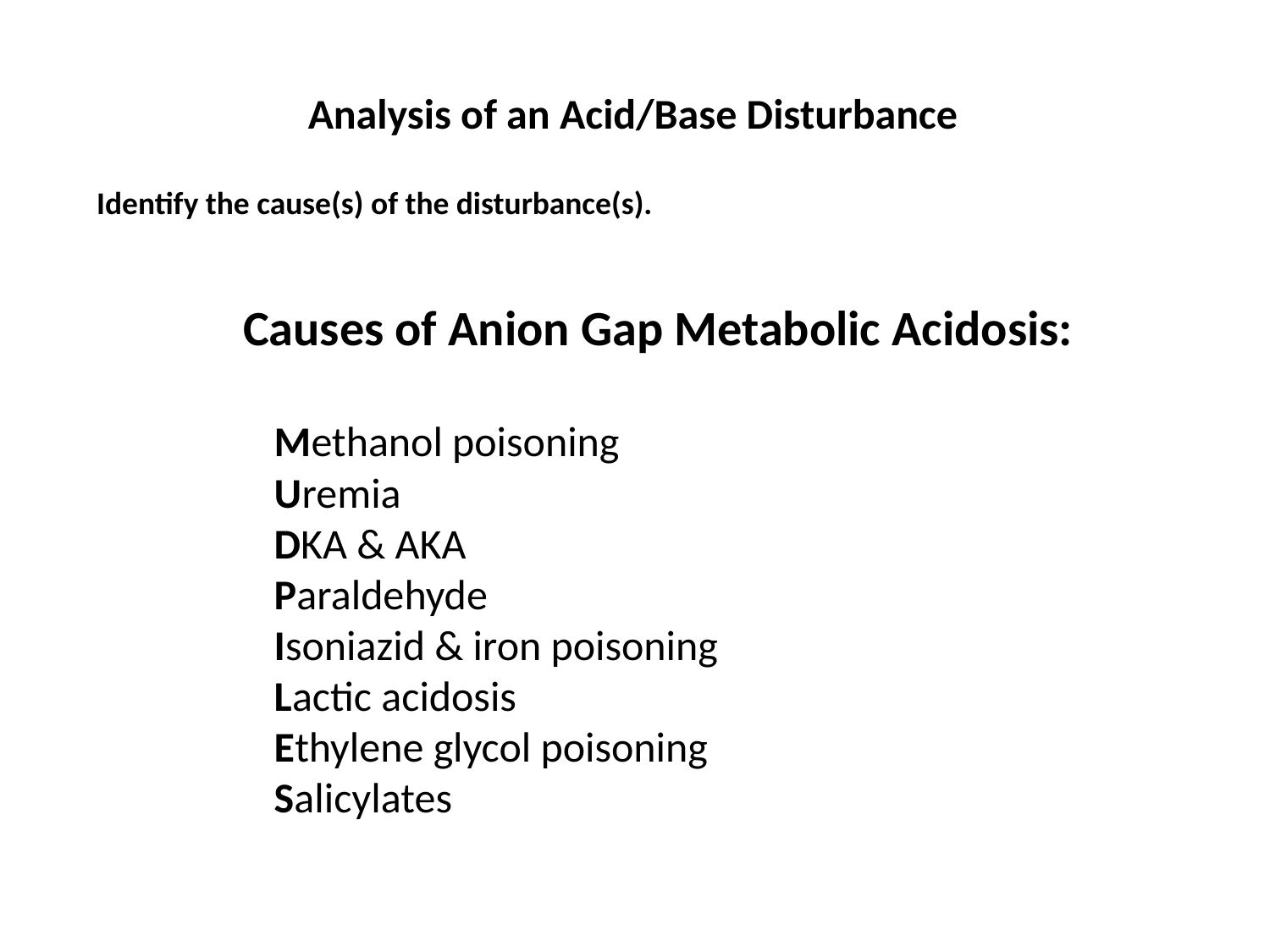

Analysis of an Acid/Base Disturbance
Identify the cause(s) of the disturbance(s).
Causes of Anion Gap Metabolic Acidosis:
	Methanol poisoning
	Uremia
	DKA & AKA
	Paraldehyde
	Isoniazid & iron poisoning
	Lactic acidosis
	Ethylene glycol poisoning
	Salicylates

## Slide 16
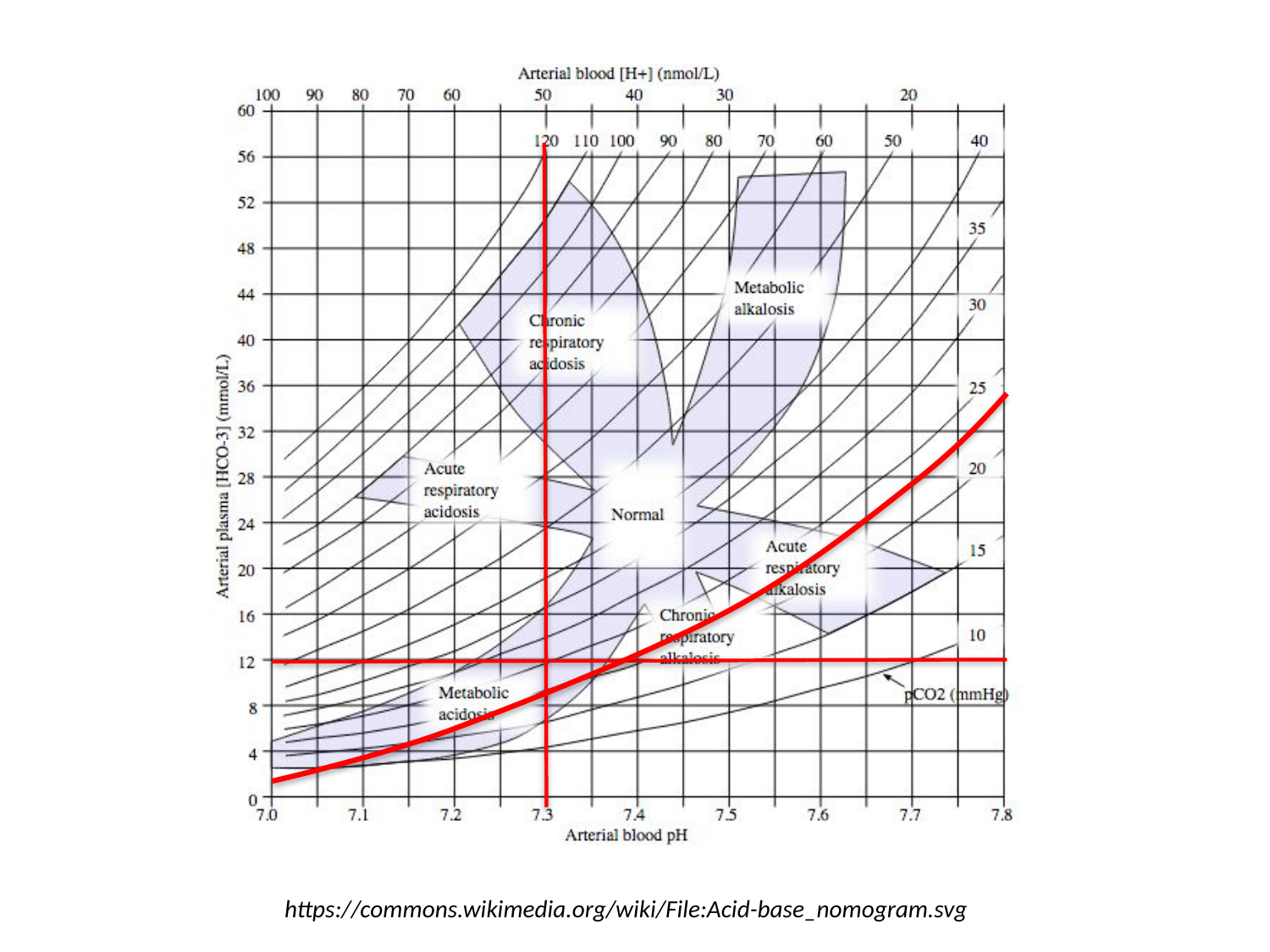

https://commons.wikimedia.org/wiki/File:Acid-base_nomogram.svg

## Slide 17
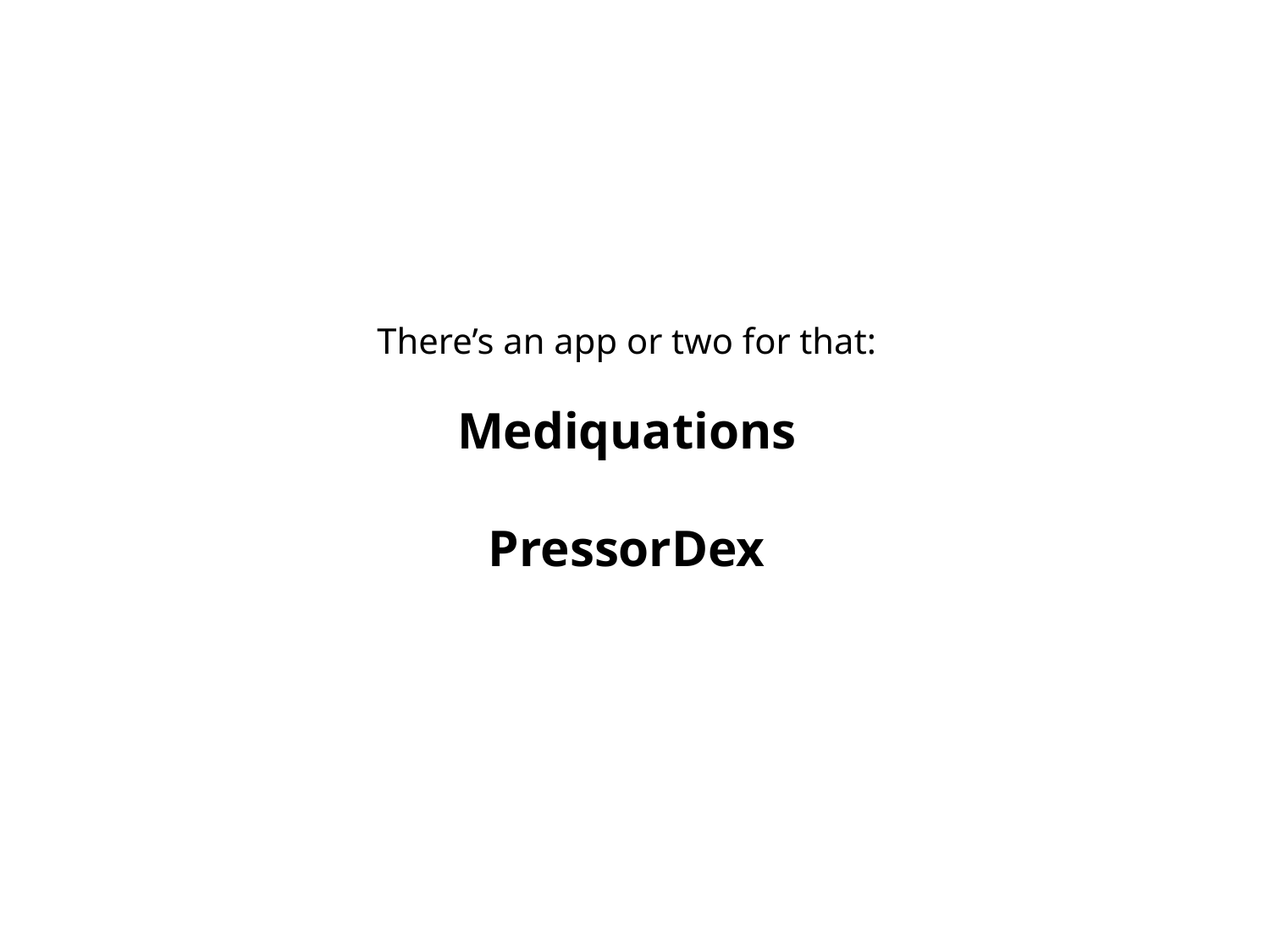

There’s an app or two for that:
Mediquations
PressorDex

## Slide 18
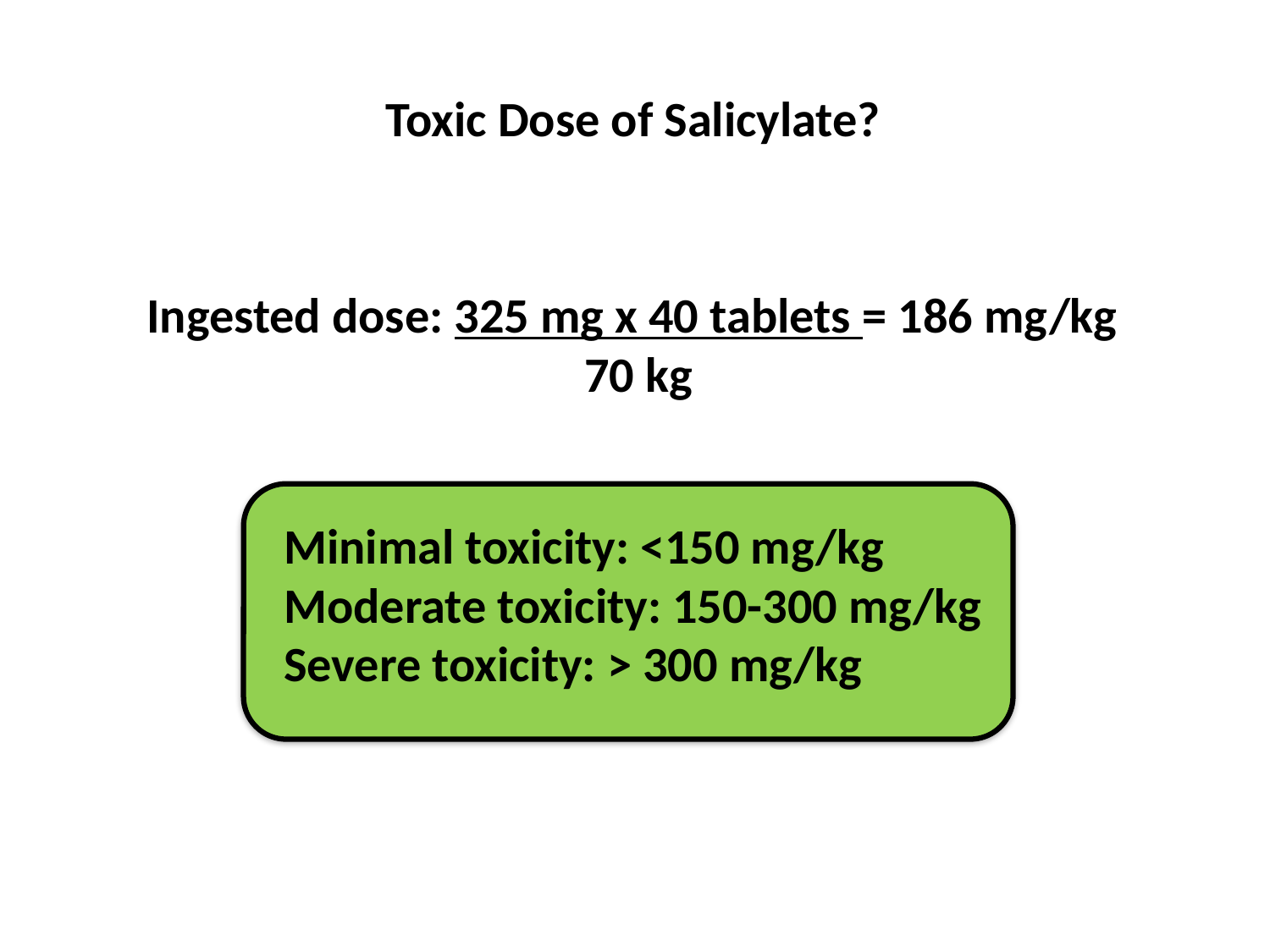

Toxic Dose of Salicylate?
Ingested dose: 325 mg x 40 tablets = 186 mg/kg
 70 kg
Minimal toxicity: <150 mg/kg
Moderate toxicity: 150-300 mg/kg
Severe toxicity: > 300 mg/kg

## Slide 19
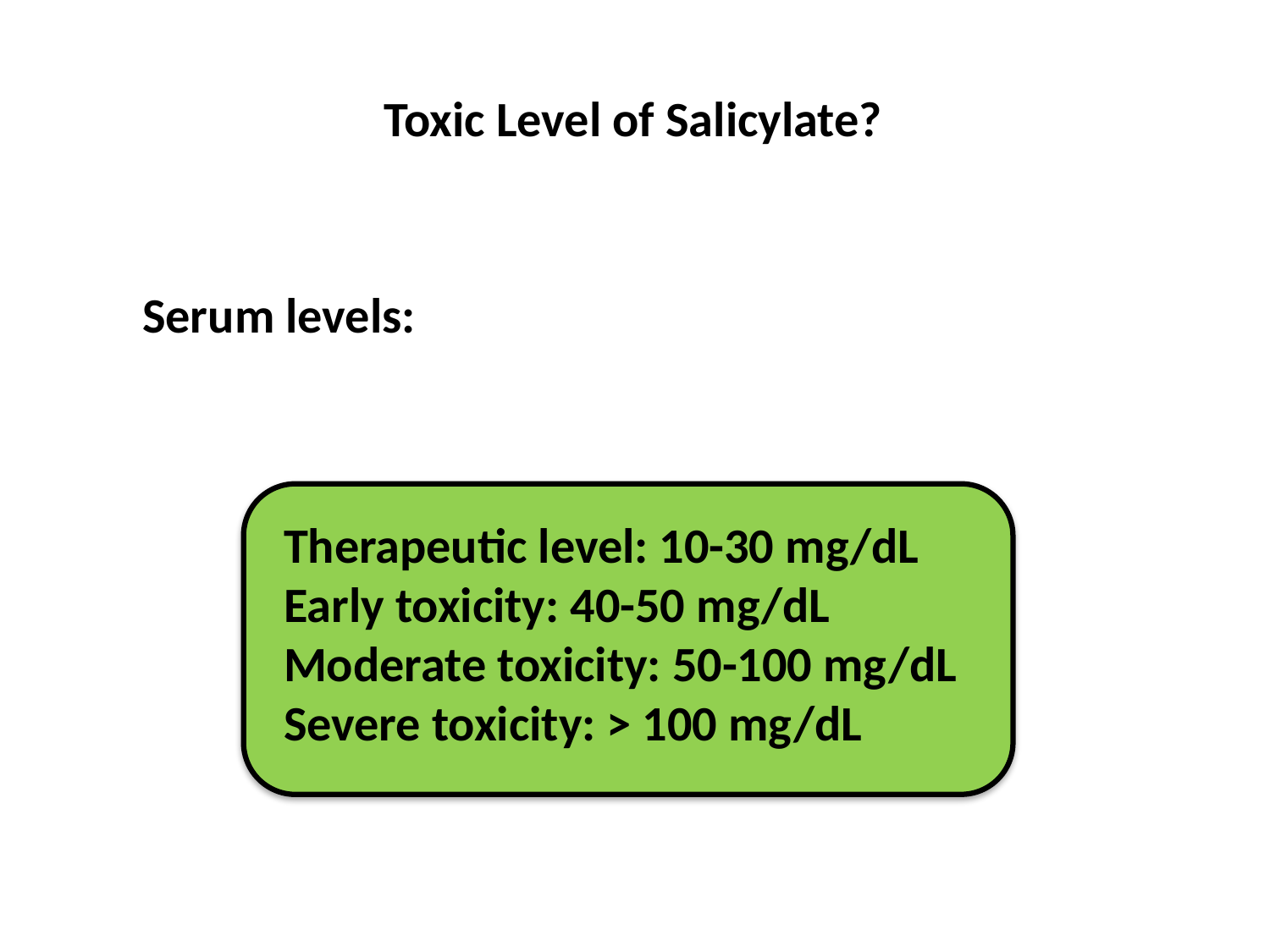

Toxic Level of Salicylate?
Serum levels:
Therapeutic level: 10-30 mg/dL
Early toxicity: 40-50 mg/dL
Moderate toxicity: 50-100 mg/dL
Severe toxicity: > 100 mg/dL

## Slide 20
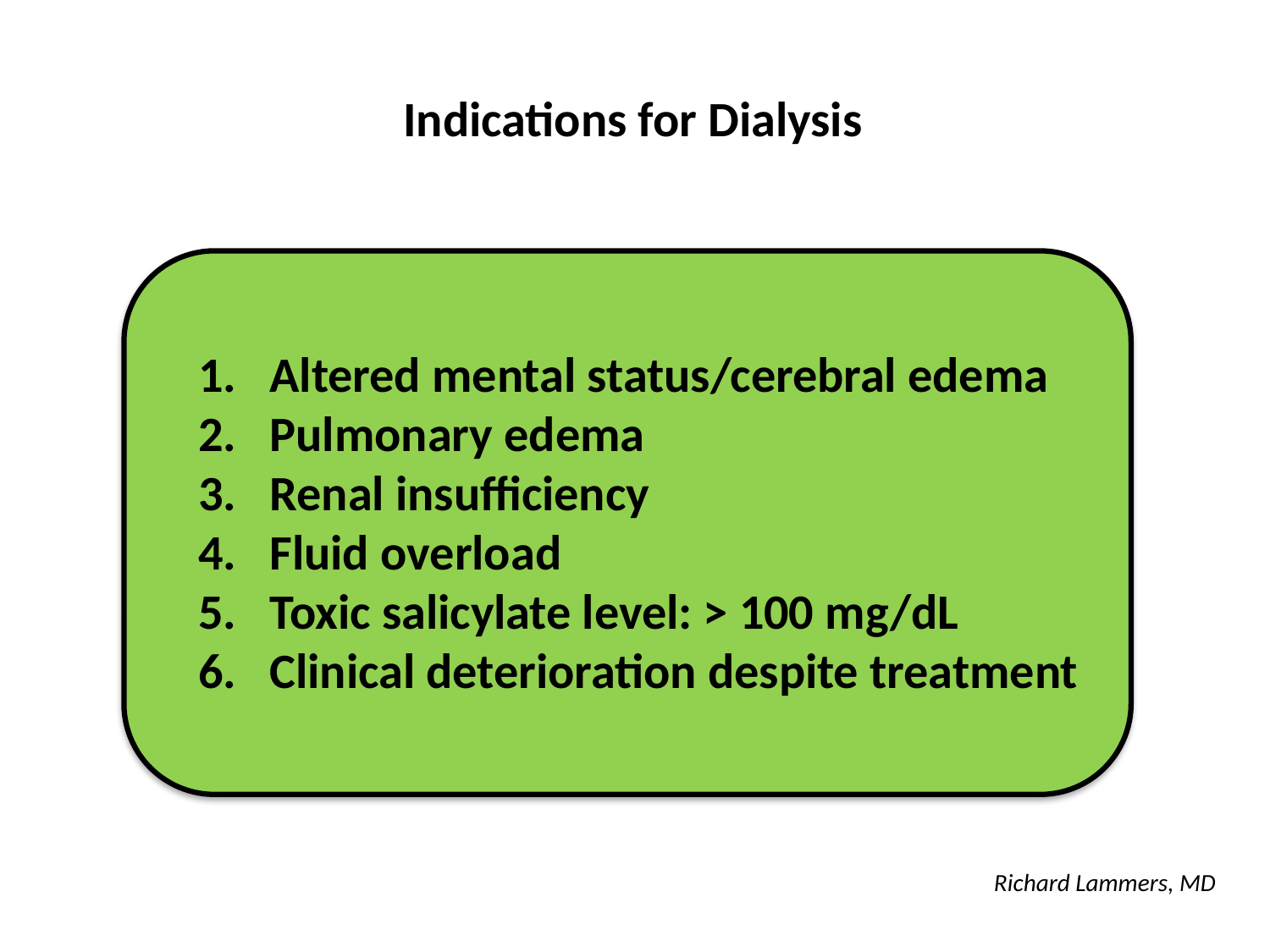

Indications for Dialysis
Altered mental status/cerebral edema
Pulmonary edema
Renal insufficiency
Fluid overload
Toxic salicylate level: > 100 mg/dL
Clinical deterioration despite treatment
Richard Lammers, MD
